# Temporal Models for Demographic and Global Health Outcomes in Multiple Populations: Introducing a New Framework to Review and Standardise Documentation of Model Assumptions and Facilitate Model Comparison

**DOI:** 10.1111/insr.12491

**Published:** 2022-03-07

**Authors:** Herbert Susmann, Monica Alexander, Leontine Alkema

**Affiliations:** ^1^ Department of Biostatistics and Epidemiology University of Massachusetts Amherst 715 North Pleasant Street Amherst MA 01003 USA; ^2^ Departments of Statistical Sciences and Sociology University of Toronto 700 University Avenue Toronto ON M5G 1Z5 Canada

**Keywords:** ARIMA processes, Bayesian methods, forecasting, Gaussian processes, hierarchical models, model documentation, temporal models, temporal models for multiple populations

## Abstract

There is growing interest in producing estimates of demographic and global health indicators in populations with limited data. Statistical models are needed to combine data from multiple data sources into estimates and projections with uncertainty. Diverse modelling approaches have been applied to this problem, making comparisons between models difficult. We propose a model class, Temporal Models for Multiple Populations (TMMPs), to facilitate both documentation of model assumptions in a standardised way and comparison across models. The class makes a distinction between the process model, which describes latent trends in the indicator interest, and the data model, which describes the data generating process of the observed data. We provide a general notation for the process model that encompasses many popular temporal modelling techniques, and we show how existing models for a variety of indicators can be written using this notation. We end with a discussion of outstanding questions and future directions.

## 1 Introduction

Population‐level measures of demographic and health indicators over time are essential to identify which populations are the most disadvantaged, where progress has been made or is stalling, and help to inform resource allocation. Important indicators include measures of health and mortality at different ages, fertility, family planning measures and migration. Assessing changes in quantities over time is useful for both cross‐country and within‐country comparisons to see how outcomes have changed in the past and how they are likely to change in future.

**FIGURE 1:**
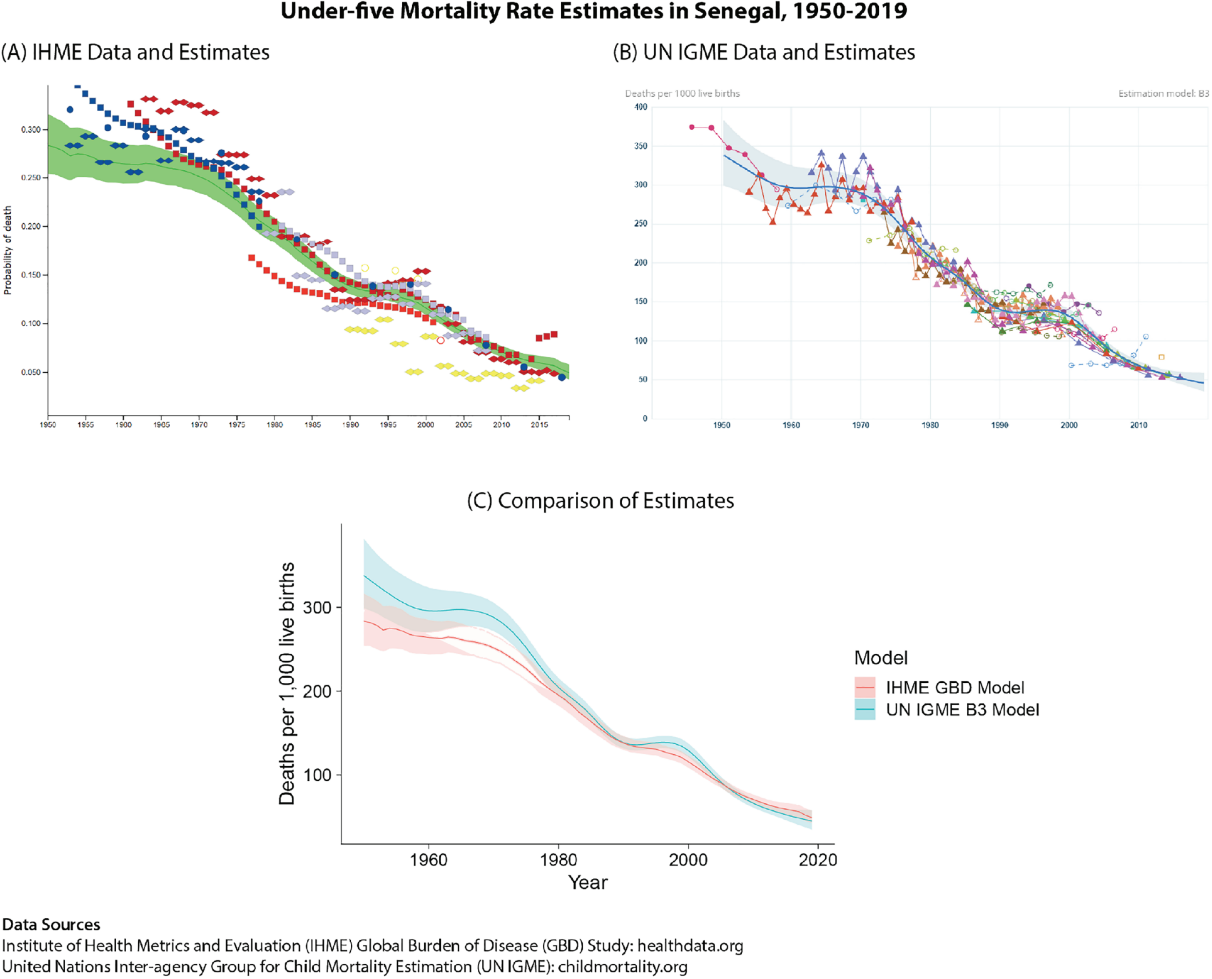
Comparison of estimates of the under‐five mortality rate in Senegal from 1950 to 2019 from the most recent estimates available from two models: the Global Burden of Disease (GBD) Model from the Institute of Health Metrics and Evaluation (IHME), and the B3 model from the UN Inter‐agency Group for Child Mortality Estimation (IGME). While both models generate estimates of the same indicator, they have different data inclusion criteria and modelling assumptions, leading to diverging estimates. (A) Raw data and model estimates from the IHME Global Burden of Disease Study; source: healthdata.org, accessed 12/22/2020. (B) Raw data and model estimates from the UN IGME B3 model; source: childmortality.org, accessed 12/22/2020. (C) Comparison of estimates from each model on the same scale [Colour figure can be viewed at wileyonlinelibrary.com]

In practice, producing estimates and projections of demographic and health indicators may not be straightforward due to the lack of good‐quality data. Well‐functioning civil registration and vital statistics systems, which are usually the most reliable source of demographic information, do not exist in most countries worldwide, making reliable data on births, deaths, population and other health outcomes difficult to obtain. This is particularly the case in low‐income countries, where the burden of mortality is often the highest. In many cases, there are missing observations over time, and in some populations, we may have no observations. Data that do exist are sometimes of very poor quality, and there may be issues reconciling different observations of the same outcome. For example, in the absence of civil registration and vital statistics systems data, large‐scale national surveys such as the Demographic and Health Surveys and Multiple Indicator Cluster Survey (Croft *et al*., 2018; Khan & Hancioglu, 2019) are common sources of information about health and mortality; however, even if the surveys cover an overlapping period, measurements derived from the two sources are not necessarily in agreement. Figure 1 illustrates this problem by showing the data used for estimating under‐five mortality rates in Senegal, which come from a variety of data sources and are often not in agreement with one another.

As such, statistical methods are usually required to model indicators over time because the data we observe are incomplete or paint an imperfect picture of what we are actually trying to estimate. Statistical models for the temporal estimation of demographic and health indicators have been used for decades, with some of the earliest efforts producing stochastic forecasts of mortality and fertility in the United States (Lee & Carter, 1992; Lee & Tuljapurkar, 1994). As computational speed and power has increased, so too has the complexity of models. In the global health context, the era of monitoring progress towards Development Goals has elicited the creation of statistical models to capture non‐linear trends in a wide range of indicators across a variety of data‐sparse situations; refer to, for example, work in estimating under‐five mortality rates (Alkema & New, 2014; You *et al*., 2015; Dicker *et al*., 2018; Li *et al*., 2019) and maternal mortality (Alkema *et al*., 2016; Alkema *et al*., 2017; Kassebaum *et al*., 2016). In addition, as the focus of studying population‐level outcomes has shifted to understanding inequalities across key subgroups, the methods used to monitor trends at the macro level have also been applied to more finer‐grained geographies, particularly where issues of small populations become apparent (Alexander *et al*., 2017; Burstein *et al*., 2019; Li *et al*., 2019). Statistical methods to account for and smooth temporal variation used are wide ranging and include ARIMA models (Alkema *et al*., 2017), penalised splines regression (Alexander & Alkema, 2018), and Gaussian process regression (Dicker *et al*., 2018). Models may be hierarchical, spatial, and include explanatory covariates.

On the outset, the statistical techniques used to model important demographic and health indicators appear to be diverse and highly dependent on the specific outcome being estimated, the background of the researchers, and the intended audience and users of the estimates. However, when viewed in the broader context of common modelling goals—to produce reliable estimates and projections over time, accounting for missing data and multiple data sources, and to give an idea of the level of uncertainty in estimates and projections—a large set of models can be explained in reference to a general model class.

In this paper, we propose such a model class, ‘Temporal Models for Multiple Populations’ (TMMPs), that encompasses many existing demographic and health models. The model class has two main components: a process model and a data model. The aim of the process model is to capture trends over time in the indicator of interest, which is itself unobserved. The process model includes systematic and covariate components, which may capture parametric trends and the relationship between the indicator and covariates, and a stochastic temporal smoothing component, which allows for data‐driven trends over time. The data model relates the observed data to the underlying latent outcome of interest, taking into account different types of error that may be present in different data sources. Considering models in this class makes clear the distinction between the goal of capturing systematic, underlying trends in the ‘true’ outcome of interest, and the goal of accounting and adjusting for different types of measurement error in observations.

We focus in this paper primarily on the form of the process model and present a general notation for process models that encompasses many existing models. We illustrate this by showing how several existing models can be seen as falling into the TMMP model class and describe in detail how their process models can be written using our notation. The first two models we describe both aim to produce country‐level estimates of the under‐five mortality rate (U5MR) but employ markedly different strategies (Alkema & New, 2014; Dicker *et al*., 2018). Other models discussed are as follows: a model of contraceptive use rates, which provides an example of a model that captures a transition process (Cahill *et al*., 2018); a model to estimate neonatal mortality rates globally, which includes a strong association with an underlying covariate (Alexander & Alkema, 2018); estimating maternal mortality rates, which combines a multilevel mixed model and an ARIMA time series model (Alkema *et al*., 2017); and a model of subnational age‐specific mortality, which shows how a more traditional demographic modelling approach can be expressed in our model class (Alexander *et al*., 2017).

The rest of this paper is structured as follows. Section 2 formalises the setting, describes the core statistical task at hand and introduces the U5MR case study. Section 3 introduces our proposed model class, TMMPs, and describes in detail each component of the process model. We then return to the U5MR case study in Section 4, showing how two existing models for the indicator can be written as TMMPs. Several other examples of existing models rewritten in TMMP notation are included in Section 5. We discuss open problems and future directions for research in Section 6.

## 2 Problem Statement

In this section, we describe the overarching statistical problem that the models in our proposed class intend to solve and introduce a case study of two existing models for estimating U5MR in countries over time. The models we consider are designed to produce estimates and future projections of a particular indicator for a set of populations over a certain time period. In general, the indicator of interest could be any population‐level demographic or health indicator. The populations of interest could include national, regional or subnational populations, or population subgroups based on some other characteristic (e.g. sex, wealth quintile or education); the only commonality is that indicators for multiple populations are being modelled within the same framework.

The time period of interest can range any possible time span but usually includes a period beyond the most recent observation year in which case projections need to be made. Long‐term projections may be of interest; for example, a common future year for projections is 2030, which is the target year for reaching the Sustainable Development Goals.

In many contexts, there may not be a complete set of observations of the outcome of interest for all time points and populations. Additionally, data sources may be of differing quality, with varying levels of random error and systematic biases. The overall goal of a model is to use the available data to produce a full time series of the outcome for all populations of interest. Models need to be able to account for these errors and biases when producing estimates for years with observations and be structured such that estimates can also be obtained for years without observations.

Formally, let t=1,…,T index the time period and c=1,…,C index the populations of interest. The goal is to estimate an indicator of interest in each population at every time point, which we denote *η*_*c*, *t*_. *η*_*c*, *t*_ may refer to a population parameter that cannot be directly observed, such as a probability or risk associated with a certain event, or to a population mean if all members of the population were sampled. The value of *η*_*c*, *t*_ is typically not observed and must be estimated from available data. The model output should accurately describe the uncertainty in the estimates of *η*_*c*, *t*_. To inform the model, there are observations $y_{i},\;i=1,\ldots ,N$ of the indicator. Each of these observations is associated with a time point *t*[*i*] and population *c*[*i*]. There may be multiple observations for the same population and time point, and there may be populations with few or no observations. Observations can come from a variety of sources and may involve preprocessing steps. There may also be estimates of error associated with each observation. For example, microdata from surveys are often preprocessed to compute estimates of a population mean, with each point estimate being accompanied by an estimate of the survey sampling error.

### 2.1 Case Study

Consider the problem of estimating U5MR in countries over time. The U5MR is one of the most important indicators of child mortality, forming the basis of one of the Millennium Development Goals and now one of the Sustainable Development Goals. In particular, Sustainable Development Goal 3.2 stipulates that by 2030, all countries should reduce U5MR to at least as low as 25 per 1000 live births. As a consequence of the focus on U5MR in the global health community, much work has been done on estimating and projecting trends in U5MR over time, as well as trying to understand these trends (Alkema & New, 2014; Dicker *et al*., 2018; United Nations Inter‐agency Group for Child Mortality Estimation (UN IGME), 2020; Li *et al*., 2019; Wang *et al*., 2017; You *et al*., 2015).

Specifically, let *η*_*c*, *t*_ be the crisis‐free U5MR in country *c* and year *t*, defined as the (synthetic period) probability that a child born in country *c* and year *t* will die before reaching age 5 if subject to age‐specific crisis‐free mortality rates present during year *t*. *η*_*c*, *t*_ is expressed as the number of deaths per 1000 live births (*η*_*c*, *t*_ ∈ [0, 1000]). The observed values *y*_*i*_ (*y*_*i*_ ∈ [0, 1000]) of the U5MR are derived from sources including vital registration systems, surveys, censuses and specialised studies. Estimates of sampling error may be available, depending on the data source; for example, design‐based estimates of sampling error may be available from surveys (Pedersen & Liu, 2012).

In terms of producing reliable estimates and projections for U5MR, one of the main challenges occurs with trying to reconcile multiple, incomplete data sources in low‐income countries, filling in gaps in the time series and giving reasonable bounds of uncertainty in estimates and projections. Two main groups have formulated models to estimate U5MR for all countries. The first is a group at the Institute of Health and Metrics Evaluation, as part of their work on the Global Burden of Disease (GBD) Study (Dicker *et al*., 2018). The second is the United Nations Inter‐agency Group on Child Mortality Estimation (UN IGME), whose model (referred to as the ‘B3’ model) forms the basis of the official U5MR estimates produced by UNICEF (Alkema & New, 2014). The models produce differing estimates of U5MR: Figure 1 compares the most recent model estimates covering 1950–2019 in Senegal as an example. Differences in the estimates could be due to differences in the data chosen for inclusion, differences in data processing or differences in model assumptions (Alkema & You, 2012). We will focus on how our proposed framework can be used to compare their modelling approaches.

With regards to their modelling choices, the two models take diverging paths. They make different choices about use of covariates and how to impose smoothness on the final estimates. For example, while the GBD model uses covariates derived from other data sources, such as income per capita and education levels, the B3 model relies solely on data‐driven trends that are smoothed using penalised B‐splines. However, it is less clear how to systematically compare the two. The GBD model is presented as a three‐stage modelling procedure, which makes it difficult to compare directly to the integrated modelling approach of the B3 model. We would like to be able to compare the assumptions each models makes about how the value of the indicator evolves and how information is shared between countries. The goal of our proposed model class for TMMPs is to aid in making such direct comparisons. We return to these models in Section 4 to show how they can both be written using TMMP notation and provide descriptions of each model using their original notation in Appendix S1 for comparison.

## 3 Temporal Models for Multiple Populations

The overall goal in this paper is to propose a framework that unifies the modelling approaches used to describe the evolution of the underlying indicator of interest over time, *η*_*c*, *t*_. A number of diverse modelling approaches have been applied to this problem. While all of the approaches we will consider generate probabilistic estimates, the way they go about it can be quite different. Without the additional structure provided by a model class, it can be difficult to compare across existing approaches.

**FIGURE 2:**
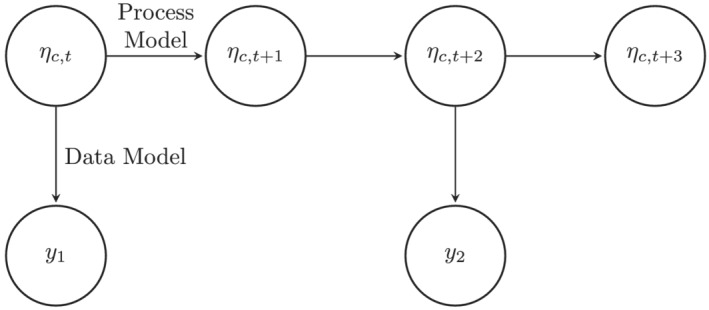
The model class distinguishes between the true values of an indicator *η*_*c*, *t*_ and the noisy observed data *y*_*i*_. The process model describes the evolution of the true values, and the data model describes how the observed data are generated from the true values. This structure handles missing data naturally: the latent trend is modelled for all time points by the process model, and it is possible that only some time points have observed values. In this example, observed data only exist for times *t* and *t* + 2

Our proposed model class, TMMPs, is organised around a distinction between observed data and latent trends. The observed data are noisy, possibly biased observations of the latent trend. Let *η*_*c*, *t*_ be the indicator of interest in population *c* at time *t*, which we emphasise is an unobserved parameter that needs to be estimated. Under this notation, modelling requires specifying a relationship between the observed *y*_*i*_ and latent *η*_*c*, *t*_, which we refer to as the data model (also often referred to in the literature as the likelihood), and describing the temporal evolution of *η*_*c*, *t*_, which we refer to as the process model (Figure 2).

As a general rule, we use Greek letters for unknown parameters and Latin letters for fixed values. For example, *σ* ^2^ for an unknown variance to be estimated, while *s* ^2^ for a fixed sampling error variance. Bold face is used to denote vectors and matrices.

### 3.1 Data Model

The data model links the observed data to the latent indicator. Let η be a *C* × *T* matrix with entries *η*_*c*, *t*_, and let $y=\{y_{1},\ldots ,y_{N}\}$ a vector of the observed values. In its most general form, the data model can be written as follows:

(1) $$y∼\pi (y|\eta ,\theta_{d})$$

where *π* is the joint distribution of y given η and a vector of parameters $\theta_{d}$. The focus of this paper is on the process model, and we leave further exploration of data models to future work. To aid understanding of their role in the model class, however, we provide several examples as illustrations for an observation indexed by *i*,  *y*_*i*_, where *c*[*i*] refers to the population of that observation and *t*[*i*] the time point of the observation:

- Normal: $y_{i}|\eta_{c[i],t[i]},\sigma_{i}^{2}∼N(\eta_{c[i],t[i]},\sigma_{i}^{2})$, where $y_{i}\in \mathbb{R}$ and $\sigma_{i}^{2}$ is the observation variance.
- Logit‐normal: $\text{logit}(y_{i})|\eta_{c[i],t[i]},\sigma_{i}^{2}∼N(\text{logit}(\eta_{c[i],t[i]}),\sigma_{i}^{2})$, where *y*_*i*_ ∈ (0, 1) and $\sigma_{i}^{2}$ is the observation variance on the logit scale.
- Binomial: $y_{i}|\eta_{c[i],t[i]}∼\text{Binom}(n_{i},\eta_{c[i],t[i]})$, where *y*_*i*_,  *n*_*i*_ are an integers.
- Negative binomial: $y_{i}|\eta_{c[i],t[i]},\phi_{i}∼\text{NegBinom}(n_{i},\eta_{c[i],t[i]},\phi_{i})$, where *y*_*i*_,  *n*_*i*_ are integers and *ϕ*_*i*_ is an overdispersion parameter.

These examples all assume that the observed data are conditionally independent given η, which is a common modelling assumption, although is not a requirement of the general data model notation. Higher level models might be placed on the parameters of the data model; for example, the overdispersion parameter in the negative binomial data model might depend on covariates or be smoothed over time. Any such models would still be considered part of the data model.

### 3.2 Process Model

The process model describes the evolution of the indicator in each population over time. TMMP process models are separated into covariate, systematic, offset and stochastic smoothing components:

(2) $$g_{1}(\eta_{c,t})=\underset{\underset{\text{component}}{\text{covariate}}}{\underbrace{g_{2}(X_{c,t},\beta_{c,t})}}+\underset{\underset{\text{component}}{\text{systematic}}}{\underbrace{g_{3}(t,\eta_{c,s\neq t},\alpha_{c})}}+\underset{\text{offset}}{\underbrace{a_{c,t}}}+\underset{\underset{\text{component}}{\underset{\text{smoothing}}{\text{stochastic}}}}{\underbrace{\epsilon_{c,t}}}$$

where

- *g*_1_ is a transformation of *η*_*c*, *t*_.
- $g_{2}(X_{c,t},\beta_{c,t})$ is the covariate component: a function of covariates *X*_*c*, *t*_ and parameter vector $\beta_{c,t}$.
- $g_{3}(t,\eta_{c,s\neq t},\alpha_{c})$ refers to a systematic temporal component: a mathematical function that describes a parametric trend over time, that may depend on prior or future values $\eta_{c,s\neq t}$ and depends on parameters $\alpha_{c}$.
- *a*_*c*, *t*_ is a deterministic, data‐driven offset that may be used to incorporate information from models estimated in a separate procedure.
- *ϵ*_*c*, *t*_ is a deviation term to allow for data‐driven deviations from the trends described by the covariate component, systematic component and the fixed offset.

The process model controls the behaviour of the model particularly in projections and data‐sparse time periods. The chosen form of the process model affects the central estimate of projections and periods with missing data and the estimated uncertainty around those point estimates. While the process model in Equation 2 is specified as a marginal model for one indicator *η*_*c*, *t*_ for population *c* and time *t*, correlation structures over time, space or other dimensions can be incorporated through the specification of the process model components. We will consider each component of the process model separately with attention to their contribution to the overall behaviour of a model.

Our proposed model class draws on the rich literature on models involving latent structures and measurement error. Measurement error models acknowledge uncertainty and bias in observed data (Carroll *et al*., 2006), as we do in our model class. State space models also make a distinction between noisy observations and an underlying latent process (Chatfield & Xing, 2019). There is a vast literature on flexible and data‐driven modelling techniques, especially for time series, which we draw on in our framework (West & Harrison, 2006). Our model class tailors these broad existing approaches to the context of demographic and health indicators by providing an overarching structure. This provides a principled way of combining the ideas behind measurement error models, state space models and smoothing models into interpretable models suited for the application of interest.

Next, we describe each process model component in more detail and provide examples of each component from existing models.

### 3.3 Covariates

In some cases, particularly in data‐sparse settings, it might make sense to include covariates in the process model, such that the indicator of interest can be modelled as a function of related factors that may have more data available. For example, there is often a strong association observed between health indicators and measures of the economy or wealth, such as a country's gross domestic product (GDP). While information on specific health indicators may be sparse, estimates of GDP are widely available.

Covariates can be included in the process model via the regression function $g_{2}(X_{c,t},\beta_{c,t})$, where $\beta_{c,t}$ are population‐level parameters. The choice of covariates to include may be informed by exploratory data analyses or substantive knowledge of the covariates that are associated or causally related to the outcome of interest. The covariate‐based component is useful for informing expected trends of indicators in projections and data‐sparse settings.

Table 1 gives examples of regression functions used in existing models of global health indicators. For example, a model to estimate maternal mortality ratios discussed in Alkema *et al*. (2017) uses GDP, general fertility rate and percentage of births with a skilled attendant present as covariates for estimating the log‐transformed proportion of non‐AIDS maternal deaths. The neonatal model discussed in Alexander & Alkema (2018) uses U5MR as a covariate, where U5MR is used as a predictor for the ratio of neonatal mortality rate (NMR) to (NMR–U5MR), that is, neonatal to other child mortality. The inclusion of U5MR as a covariate is based on the strong demographic relationship that in general, as child mortality decreases, the share of deaths that are neonatal increases. The form of this relationship is piecewise linear, based on the results of an exploratory data analysis. The GBD U5MR model of Dicker *et al*. (2018) uses a non‐linear regression function incorporating lag‐distributed income per capita (LDI), mean years of education for women age 15–49 (EDU), and the HIV death rate in ages 0–4 (HIV). Finally, the age‐specific mortality model of Alexander *et al*. (2017) uses the principle components of mortality schedules as covariates.

**TABLE 1:** Selected examples of covariate functions

Indicator	*η*_*c*, *t*_	*g*_1_(·)	*g*_2_(·)	Covariates
Maternal mortality ratio (Alkema *et al*., 2017)	Proportion of non‐AIDS deaths that are maternal	log	$\beta_{c,0}+\underset{k}{\sum }X_{c,t,k}\beta_{k}$	log(GDP), log(GFR) and SAB
Neonatal mortality rate (Alexander & Alkema, 2018)	NMR/(U5MR‐NMR)	logit	$\beta_{0,c}+\beta_{1}\cdot \left(\log(X_{c,t})-\log(\beta_{2})\right)1_{\left[X_{c,t}>\beta_{2}\right]}$	U5MR
Subnational mortality (Alexander *et al*., 2017)	Age‐specific mortality	log	$\underset{k}{\sum }X_{c,t,k}\beta_{c,t,k}$	Principal components of mortality schedule
U5MR (Dicker *et al*., 2018)	U5MR		$\log_{10}(\exp[\beta_{1}\cdot \log(X_{c,t}^{LDI})+\beta_{2}\cdot X_{c,t}^{EDU}+\beta_{3}]+\beta_{4}x_{c,t}^{HIV})$	LDI, EDU and HIV

### 3.4 Systematic Component

We may expect the trend over time of some demographic and health indicators to follow a certain path that can be expressed as a parametric function. For example, demographic transition theory suggests that as a country's fertility rate declines, it will first decline rapidly, and then decelerate, and eventually plateau at a certain level (Kirk, 1996). A similar assumption about rates of change can be made for contraceptive prevalence over time. Such assumptions can be incorporated by a mathematical function $g_{3}(t,\eta_{c,s\neq t},\alpha_{c}),$ which encodes a parametric trend depending on time *t*, past or future values of the indicator $\eta_{c,s\neq t}$, and a set of parameters $\alpha_{c}$. The form of this function may be informed by prior knowledge of the mechanisms that drive the evolution of the indicator of interest.

Table 2 gives examples of systematic components used in a selection of existing models. For example, the process model of Cahill *et al*. (2018) describes the total contraceptive use rate in a country over time. Based on a modelling assumption that a country's total contraceptive use rate will increase slowly, speed up and then taper off as it moves through the contraceptive use transition, their model includes a systematic component that captures an expected rate of change based on a logistic growth curve.

**TABLE 2:** Selected examples of systematic components

Indicator	*g*_3_(·)
Total contraceptive use (Cahill *et al*., 2018)	Logistic curve:
	$g_{3}(\cdot )=\Omega_{c}$ when $t=t^{*}$, and for *t* > *t* ^∗^:
	$g_{3}(\cdot )=\text{logit}\left(\eta_{c,t-1}\right)+\iota_{c,t}$,
	= $\left\{\begin{array}{cc} \text{logit}\left({\tilde{P}}_{c}\cdot {\text{logit}}^{-1}\left(\text{logit}\left(\frac{\eta_{c,t-1}}{{\tilde{P}}_{c}}\right)+\omega_{c}\right)\right),\; & \;\text{when}\;\eta_{c,t-1}<{\tilde{P}}_{c}\\ \;\text{logit}\left(\eta_{c,t-1}\right),\; & \text{otherwise,} \end{array}\right.$
	where $\alpha_{c}=\left\{{\tilde{P}}_{c},\omega_{c},\Omega_{c}\right\}$.
Inflation in the sex ratio at birth (Chao *et al*., 2019)	Trapezoid function:
	$\left\{\begin{array}{cc} \xi_{c}(t-\gamma_{0,c})/\lambda_{1,c},\; & \gamma_{0,c}<t<\gamma_{c,1}\\ {\;\xi }_{c},\; & \gamma_{1,c}<t<\gamma_{2,c}\\ \xi_{c}-\xi_{c}(t-\gamma_{2,c})/\lambda_{3,c},\; & t<\gamma_{2,c}<t<\gamma_{3,c}\\ \;0,\; & \text{otherwise} \end{array}\right.$ where $\alpha_{c}=\left\{\xi_{c},\gamma_{0,c},\lambda_{c,1},\lambda_{2,c}\right\},\;\gamma_{c,1}=\gamma_{c,0}+\lambda_{c,1},\;\gamma_{2,c}=\gamma_{c,1}+\lambda_{2,c}$, and $\gamma_{3,c}=\gamma_{2,c}+\lambda_{3,c}$.

### 3.5 Offset

Some models incorporate information from separate modelling stages into their process model. The TMMP model class accounts for this through the *a*_*c*, *t*_ term in the process model. We chose to separate this term into a separate component to make it clearer when models incorporate fixed external information besides covariates. In particular, the offset might be derived from the observed data y in some way, meaning the data are used twice (once in the offset, to specify η, and once in the main TMMP data model.) For example, in the GBD U5MR model, the observed U5MR data y is used in the TMMP data model as well as to calculate the offsets. The offsets are derived from the smoothed residuals from a regression model fitted to the observed data and serves to adjust the output of the covariate component (Dicker *et al*., 2018).

### 3.6 Stochastic Smoothing Component

The final part of the process model, the stochastic smoothing component, captures trends that are not explained by the systematic component, covariate component or offset. To prevent overfitting, a model is placed on the deviations to enforce some degree of smoothness in the model fit. We define a smoothing model as the distribution placed on the joint vector of all deviations from a population, $\epsilon_{c}=[\epsilon_{c,1},\ldots \;,\epsilon_{c,T}]$. The smoothing model is defined as follows:

(3) $$\epsilon_{c}=B_{c}\delta_{c},$$

where $B_{c}$ is a *T* × *K*_*c*_,  *K*_*c*_ ≤ *T* full rank matrix and $\delta_{c}=[\delta_{c,1},\delta_{c,2},\ldots ,\delta_{c,K_{c}}]$ is a vector of parameters.

We define the parameter vector $\delta_{c}$, or its differenced version, to be normally distributed with mean zero: for *r* ≥ 0

(4) $${△}_{r}\delta_{c}|\Sigma_{c}∼N(0,\Sigma_{c}),$$

where ${△}_{r}$ is the difference operator with ${△}_{1}\delta =[\delta_{2}-\delta_{1},\ldots ,\delta_{K_{c}}-\delta_{K_{c}-1}]$ and ${△}_{r}\delta ={△}_{1}^{r}\delta$. When *r* > 0, to ensure the model is generative, we need to introduce extra model structure to anchor the overall level of the deviations. Formally, we require

(5) $$\underset{k\in K_{d,c}}{\sum }{△}_{d}\delta_{c,k}=0,\text{for}\;0\leq d\leq (r-1),$$

for set of indexes $K_{d,c}$.

The covariance matrix $\Sigma_{c}$ is specified via an autocovariance function *s*, which depends on a vector of hyperparameters κ. We restrict the set of autocovariance functions to those that can be expressed as a function only of the absolute distance between *t*_1_ and *t*_2_:

(6) $$\Sigma_{c,t_{1},t_{2}}=s(|t_{1}-t_{2}|,\kappa ).$$

We further require that the covariance kernel goes to zero as |*t*_1_ − *t*_2_| goes to infinity. Commonly used autocovariance functions in this situation are the autocovariance associated with an autoregressive process of order 1 (AR(1) process), the squared exponential covariance function and the Matérn covariance function, which have the following forms:

(7) $$s_{\mathrm{AR1}}(|t_{1}-t_{2}|,\kappa )=\kappa^{2}\rho^{\left|t_{1}-t_{2}\right|}\;\text{with}\;\kappa =\{\kappa ,\rho \},$$

(8) $$s_{\text{SE}}(|t_{1}-t_{2}|,\kappa )=\kappa^{2}\exp\left(-\frac{{\left(t_{1}-t_{2}\right)}^{2}}{2\ell^{2}}\right)\;\text{with}\;\kappa =\{\kappa ,\ell \},$$

(9) $$s_{\mathrm{Matérn}}(|t_{1}-t_{2}|,\kappa )=\kappa^{2}\frac{2^{1-\nu }}{\Gamma (\nu )}{\left(\sqrt{2\nu }\frac{\left|t_{1}-t_{2}\right|}{\ell }\right)}^{\nu }K_{\nu }\left(\sqrt{2\nu }\frac{\left|t_{1}-t_{2}\right|}{\ell }\right)\;\text{with}\;\kappa =\{\kappa ,\ell ,\nu \},$$

where Γ is the gamma function and *K*_*ν*_ is the modified Bessel function of the second kind.

Smoothing models that include a transformation (i.e. $B_{c}\neq I$) include B‐spline approaches (Eilers & Marx, 1996). The transformation $B_{c}$ can be used to lower the dimension of the smoothing model; for B‐splines, for example, knots could be placed on a sparse grid in order to reduce the number of smoothing parameters to estimate. In this set up, the smoothing parameters $\delta_{c}$ are the coefficients of the spline basis functions. Because the smoothing model is the linear combination of differentiable functions, the smoothing model itself will be differentiable, which may not be the case for other choices of the smoothing model. Differentiability may be a desirable property because it implies a degree of smoothness for the smoothing model.

This definition of smoothing models as summarised in Equations (3), (4), (5) and (6) encapsulates main methods of smoothing, including ARIMA processes, random walks, Gaussian processes and B‐splines, as illustrated in Table 3.

**TABLE 3:** Selected examples of smoothing processes

Indicator	B	$s(|t_{1}-t_{2}|,\kappa )$	*r*	$K_{d,c}$
Maternal mortality ratio (Alkema *et al*., 2016)	B=I	ARMA(1, 1)	1	{1990}
U5MR (Alkema & New, 2014)	$B_{c,t,k}=b_{c,k}(t)$= cubic B‐splines, knots 2.5 years	indep. $s(|t_{1}-t_{2}|)=\sigma_{\delta ,c}^{2}1(|t_{1}-t_{2}|=0)$	2	$K_{0,c}=\left\{k_{c}^{*}\right\}$, $K_{1,c}=\left\{2,\ldots \;,K_{c}\right\}$
U5MR (Dicker *et al*., 2018)	B=I	Matérn, refer to Equation 9	0	·

When r=0, the smoothing model is stationary, in that the unconditional first and second moments of $\epsilon_{c}$ will not depend on time. When *r* > 0, however, $\epsilon_{c}$ will not be stationary, and we require the constraint

$$\underset{t\in K_{d,c}}{\sum }{△}_{d}\delta_{c,k}=0,\text{for}\;0\leq d\leq (r-1),$$

for a set of indexes $K_{d,c}$. This requirement makes the model identifiable by anchoring the sum of the undifferenced deviations at zero over the set $K_{d,c}$. If the smoothing model does not perform any dimensionality reduction by its choice of B, then $K_{d,c}$ is interpretable as the set of years for which the deviations sum to zero. Common choices include $K_{d,c}$ fixed at a specific reference year, that is, as used for family planning estimation (Cahill *et al*., 2018) and maternal mortality (Alkema *et al*., 2017). $K_{d,c}$ might also be chosen to span the observation period of the population of interest. For the models used in neonatal and child mortality estimation, the sum of the spline coefficients are constrained to sum to zero, corresponding to $K_{d,c}=\left\{1,\ldots ,K_{c}\right\}$ where *K*_*c*_ are the number of splines in population *c* (Alexander & Alkema, 2018; Alkema & New, 2014).

The behaviour of the smoothing model may influence the trends and uncertainty of projections. Appendix S2 provides further detail on stationary and non‐stationary smoothing models, including how they behave in projections.

### 3.7 Projections

It is commonly of interest to produce estimates beyond the last observed data points. Suppose the last data point in a population is observed at *T*, and we would like to project the value of the indicator to time *T* ^∗^. Using the estimation process model for projections is taken as default in the TMMP framework. In particular, based on the estimation model, *η*_*c*, *t*_ can be estimated for 1 < *t*≤*T* ^∗^ as part of the joint estimation process of the entire model. Alternatively, *η*_*c*, *t*_ can be estimated up until *T*, the last available data point, and the estimation process model can be used afterwards to generate projections from *T* to *T* ^∗^. We make explicit how projections come about for TMMP models at the end of this section.

In some modelling approaches, the projection model used for *η*_*c*, *t* > *T*_ may differ from the estimation model. This might be done to improve predictive performance and/or to investigate how different scenarios influence projections. For example, the B3 model of U5MR adopts a modified projection model that serves to control the variance in projections, improving performance in predictions (Alkema & New, 2014). The GBD Study used a different modelling procedure for projections than was used for estimating historical trends to generate mortality, fertility and migration projections to the year 2100 under various scenarios (Vollset *et al*., 2020). For such models, the projection model used needs to specified separately, either as a new TMMP model, or in terms of how the model differs from the estimation model.

#### 3.7.1 Default projections obtained from temporal models for multiple populations models

Projections require extending each model component from time *T* to time *T* ^∗^. If the covariate component uses time‐varying covariates, then this requires projected values of each covariate from *T* to *T* ^∗^. In some cases, covariate projections may already be available from a separate source. For example, the neonatal mortality model of Alexander and Alkema (2018) uses U5MR estimates and projections from the UN IGME. In cases where covariates projections are not already available, covariates need to be projected. How to do this is outside the scope of this paper but could involve building separate TMMP‐style models for the covariates.

For TMPPs with smoothers, the TMMP projection process model defaults to the *r*th‐order differenced estimation process model. For example, if the smoothing model has r=0, then the projection model is given directly by the estimation model with the extended smoothing term as per Equation (13). If r=1 (the smoothing model has one level of differencing), then the projections are based on the first‐order differences of the process model. To illustrate, consider a process model that includes a covariate component, systematic component and stochastic smoothing component with r=1. The process model is written

(10) $$\eta_{c,t}=g_{2}(X_{c,t},\beta_{c,t})+g_{3}(t,\eta_{c,s\neq t,\alpha_{c}})+\epsilon_{c,t},$$

where $\epsilon_{c}=B_{c}\delta_{c}$.

The first‐order difference of the process model is given by

(11) $$\begin{array}{cc} \eta_{c,t}-\eta_{c,t-1}= & \;(g_{2}(X_{c,t},\beta_{c,t})-g_{2}(X_{c,t-1},\beta_{c,t}))\;\\ & \;+(g_{3}(t,\eta_{c,s\neq t},\alpha_{c})-g_{3}(t-1,\eta_{c,s\neq t-1},\alpha_{c})\;\\ & \;+(\epsilon_{c,t}-\epsilon_{c,t-1}).\; \end{array}$$

The first‐order differences *ϵ*_*c*, *t*_ − *ϵ*_*c*, *t* − 1_ are multivariate normally distributed, and the conditional distribution of the first‐order differences (*ϵ*_*c*, *t*_ − *ϵ*_*c*, *t* − 1_) in the projection period then follows from Equations (13) and (14).

Next, we describe how the smoothing component can be projected. The stochastic smoothing component up to time *T* ^∗^ is given by

(12) $$\epsilon_{c}=B_{c}\delta_{c},$$

where $\epsilon_{c}$ is a *T* × 1 vector, $B_{c}$ is a *T* × *K*_*c*_ matrix and $\delta_{c}$ is a *K*_*c*_ × 1 vector. To extend the stochastic smoothing component to the end of the projection period *T* ^∗^, we define an extended vector of smoothing terms $\epsilon_{c}^{*}$ with

(13) $$\epsilon_{c}^{*}=B_{c}^{*}\delta_{c}^{*},$$

where $\epsilon_{c}^{*}$ is a *T* ^∗^ × 1 vector, $B_{c}$ is a $T^{*}\times K_{c}^{*}$ matrix and $\delta_{c}$ is a $K_{c}^{*}\times 1$ vector. Note that in a B‐spline set up, this likely includes adding extra knots to cover the projection period, which corresponds to adding additional columns to $B^{*}$. When the projection uses the estimation process model, we have that

(14) $${△}_{r}\delta_{c}^{*}|\Sigma_{c}^{*}∼N\left(0,\Sigma_{c}^{*}\right).$$

The distribution of the (differenced) smoothing terms in the projection period follows from the conditional distribution ${△}_{r}\delta_{c,t>T}^{*}|\delta_{c},\Sigma_{c}$, which has a closed form.

### 3.8 Parameter Estimation

The TMMP framework requires estimating many population‐level parameters. In the process model the systematic trend depends on $\alpha_{c}$, the covariate function depends on $\beta_{c,t}$, and the smoothing model may have hyperparameters κ. To complete the specification of a model, we need to define how these parameters will be estimated. In this section, we describe several approaches, including fixing parameters, use of informative priors and hierarchical modelling, and present examples of how they have been used in existing models. These approaches can be easily combined within a model: for example, informative priors might be set for some parameters while hierarchical distributions are used for others. Documentation of the assumptions made is important to make explicit the sharing of information, for example, across populations, the use of external information to inform the estimates or reuse of data multiple times.

#### 3.8.1 Fixed parameters

Parameters can be fixed by the modeler to set values based on substantive knowledge, convenience or results from separate models. For example, Dicker *et al*. (2018) set the country‐level hyperparameters of their smoothing model to fixed values depending on a measure of data availability in each country. They also use fixed regression parameters in the covariate term in the process model; these regression coefficients are obtained from fitting a regression model to a global data set. The offset terms *a*_*c*, *t*_ are obtained from smoothing the residuals of that regression.

#### 3.8.2 Priors

If the model is fit in a Bayesian inferential framework, then prior distributions can be applied to parameters to inform estimation. When substantive information is available for parameters, then informative priors can be used. Vague priors can be used when there is little or no prior knowledge to inform the parameter values. The models considered in this paper as examples use vague priors for most model hyperparameters. We note that informative priors have been used more often for data bias and measurement error parameters in data models, a discussion of which we leave to future work.

#### 3.8.3 Hierarchical modelling

Hierarchical (or multilevel) modelling is a general approach that can be used for parameter estimation when it is desirable to share information between units. In such a set up, unit‐specific parameters can be estimated in different ways according to how the modeler wants to share information between units, often referring to populations in TMMPs. At one extreme, parameters could be estimated independently for each population. This encodes an assumption that the parameter in one population is unrelated to the parameter in another population. Another extreme is to have every population share the same set of parameters, which is appropriate for modelling global level patterns that are shared across all populations. In between these two options is an intermediary choice in which information is shared between populations via hierarchical modelling. By using hierarchical modelling, the parameter estimates in one population can be informed by parameter estimates in similar populations. Sharing information can be especially useful in cases where some populations have limited or no data available, but the modeler still wishes to generate estimates.

To ease comparison between models, we introduce a basic notation for hierarchical models, which encodes distributional assumptions and the levels and groupings of countries. For a population‐level parameter *γ*_*c*_, which may be defined on a transformed scale and can belong to any of the process model components, a hierarchical model can be written as

$$\gamma_{c}∼\pi (\gamma_{c}|\gamma_{r[c]}^{\left(region\right)},\sigma_{\gamma }^{(region)2}),$$

where *π* represents a probability distribution. The parameters $\gamma_{r[c]}^{\left(region\right)}$ are the group‐level means, where *r*[*c*] indexes the group that population *c* belongs to. The parameter $\sigma_{\gamma }^{(region)2}$ is the variance of the population‐level parameters around their group means. This set up can be extended recursively to allow for deeper hierarchies, where the group *r*[*c*] can be a member of a higher level group itself, and so forth. Specifying a hierarchical model therefore requires defining the distribution *π*, the number of levels in the hierarchy and the hierarchical groupings. A common set up is to take *π* to be the normal distribution and to have only one level of hierarchy, in which case r[c]=1 for all *c* and $\gamma_{1}^{\left(region\right)}$ represents a global mean:

$$\gamma_{c}|\gamma^{\left(world\right)},\sigma_{\gamma }^{2}∼N(\gamma^{\left(world\right)},\sigma_{\gamma }^{2}).$$

The use of hierarchical modelling in several models is compared in Table 4, giving their choices for distribution *π* and the number of levels and hierarchical groupings.

**TABLE 4:** Hierarchical modelling in selected models

Indicator	Hierarchical models	*π*	Levels	Groupings
Total contraceptive use (Cahill *et al*., 2018)	Asymptote $\tilde{P}$	Normal	1	Countries within world
	Rate *ω*_*c*_	Normal	3	Countries within subregion, region, world
	Timing Ω_*c*_, developing countries	Normal	3	Countries within subregion, region, world
	Timing Ω_*c*_, developed countries	Normal	1	Countries within world
Maternal mortality (Alkema *et al*., 2017)	Smoothing parameters *λ*_*c*_	Truncated normal	1	Countries within world
Age‐specific mortality (Alexander *et al*., 2017)	Regression coefficients *β*_*k*, *a*_	Normal	1	Counties within state

### 3.9 Inference and Computation

The TMMP model class is agnostic to the choice of inferential framework. However, the use of hierarchical modelling for estimating TMMP parameters lends itself naturally to Bayesian inference. If Bayesian inference is chosen, probabilistic programming languages such as Stan or JAGS can be used to specify TMMP models and sample from the joint posterior distribution of the model parameters (Stan Development Team, 2019; Plummer, 2003). Approximate methods such as integrated nested Laplace approximations can be used as long as TMMP qualifies as a latent Gaussian field (Rue *et al*., 2009). To perform frequentist inference, the Template Model Builder framework (Kristensen *et al*., 2016) can fit a similar class of models as integrated nested Laplace approximations, with similar results (Osgood‐Zimmerman & Wakefield, 2021).

### 3.10 Temporal Models for Multiple Populations Reporting Template

To standardise the writing of model assumptions for TMMPs, including those related to parameter estimation, we propose the use of TMMP reporting templates. We provide a template in Appendix S3 and illustrate its use for the case studies discussed in the remainder of this paper.

## 4 Case Study

In this section, we describe the process models for two existing models of the U5MR in countries over time, using TMMP notation. For both models, let *η*_*c*, *t*_ be the population crisis‐free U5MR in country *c* and year *t*. That is, *η*_*c*, *t*_ is the (synthetic period) probability a child born in country *c* and year *t* will die before age 5 if subject to age‐specific crisis‐free mortality rates present during year *t*, expressed in deaths per 1000 live births. Next, we describe the two process models using TMMP notation. The process model descriptions for both models are summarised in the TMMP template in Table 5. In Appendix S1, we provide descriptions of each model using the notation in which they were originally presented.

**TABLE 5:** Comparison of the U5MR process model and estimation strategies in the IHME GBD model and the UN IGME B3 model

	GBD	B3
*η*_*c*, *t*_	U5MR	U5MR
*g*_1_(·)	$\log_{10}$	log
Process model formula	$g_{1}(\eta_{c,t})=g_{2}(X_{c,t},\beta )+a_{c,t}+\epsilon_{c,t}$	$g_{1}(\eta_{c,t})=g_{3}(t,\alpha_{c})+\epsilon_{c,t}$
Covariate component		
*g*_2_(·)	non‐linear regression formula (Equation 16)	·
Covariates	LDI, EDU, HIV	·
Systematic component		
*g*_3_(·)	·	$\alpha_{c,0}+\alpha_{c,1}(t-t_{c}^{*})$, with $t_{c}^{*}\approx$ middle of observation period
$\;\alpha_{c}$	·	intercept *α*_*c*, 0_ and slope *α*_*c*, 1_
Offsets		
*a*_*c*, *t*_	offsets obtained from smoothed residuals of a mixed‐effects regression model fit	·
Stochastic smoothing component $\epsilon_{c}=B_{c}\delta_{c}$		
B	B=I	$B_{c,k}=$ cubic B‐splines, knots every 2.5 years
*s*(*t*_1_, *t*_2_)	Matérn	indep. $s(t_{1},t_{2})=\sigma_{\delta ,c}^{2}1(t_{1}=t_{2})$
*r*	0	2
$K_{d,c}$	·	$K_{0,c}=\left\{k^{*}\right\},\;K_{1,c}=\left\{2,\ldots \;,K_{c}\right\}$
Projections (if not defaulting to estimation model)		
Projections	·	Logarithmic pooling approach: for projections, $\begin{array}{cc} {△}_{2}\delta_{c,k}∼\; & N\left(\Gamma_{c,k},\Theta_{c,k}\right),\\ \Gamma_{c,k}=\; & W\cdot G+(1-W)\cdot {△}_{2}\delta_{c,k-1},\\ \Theta_{c,k}=\; & W\cdot V+(1-W)\cdot \Theta_{c,k-1}. \end{array}$
Parameter estimation		
Fixed	Matérn covariance hyperparameters; β in covariate component	For projections, *G* and *V* equal to the median and variance of the estimates of past 2nd‐order differences ${\hat{△}}_{2}\delta_{c,k}$'s respectively, *W* fixed through validation exercise
Vague priors	·	systematic parameters *α*_*c*, 0_, *α*_*c*, 1_
Informative prior	·	·
Hierarchical distribution	·	Smoothing parameters $\sigma_{\delta ,c}^{2}$
Distribution *π*	·	Normal
Number of levels in hierarchy	·	1
Hierarchical groupings	·	Countries within world

### 4.1 Global Burden of Disease Model

#### 4.1.1 Process model

The GBD process model can be understood as having a covariance component, fixed offset and a Gaussian process smoothing model component. The process model is given by

(15) $$\log_{10}\left(\eta_{c,t}\right)=g_{2}(X_{c,t},\beta_{c})+a_{c,t}+\epsilon_{c,t}.$$

#### 4.1.2 Covariate component

The covariate component is a non‐linear function of LDI, mean years of education for women of reproductive age (15–49 years), and HIV death rate in ages 0–4 as covariates:

(16) $$g_{2}(X_{c,t},\beta )=\log_{10}\left[\exp\left[\beta_{1}\cdot \log(X_{c,t}^{\text{LDI}})+\beta_{2}\cdot X_{c,t}^{\text{EDU}}+\beta_{3}\right]+\beta_{4}x_{c,t}^{\text{HIV}}\right].$$

The coefficients are the same for every country and time point, so to simplify the notation we write β rather than $\beta_{c,t}$.

#### 4.1.3 Offset

The offset *a*_*c*, *t*_ adjusts the values from the covariate component based on the results of a separate procedure. We provide an outline here of this procedure, and a more detailed explanation can be found in the description of the first and second modelling stages in Appendix S1.

First, a mixed‐effects model is fitted to the observed data, including random effects for each data source and data source type. The fitted model is used to generate bias‐adjusted data points ${\hat{y}}_{i}^{\text{adjusted}}$ and to generate predictions of the indicator in every year and country, which we denote ${\hat{\eta }}_{c,t}^{\text{predicted}}$ where ${\hat{\eta }}_{c,t}^{\text{predicted}}=10^{g_{2}(X_{c,t},\hat{\beta })}$, with *g*_2_ defined in Equation 16. In this modelling stage, residuals are defined as the difference between the bias‐adjusted data points and the predictions; $r_{i}={\hat{y}}_{i}^{\text{adjusted}}-{\hat{\eta }}_{c[i],t[i]}^{\text{predicted}}$.

In the second modelling stage, a set of smoothed residuals ${\hat{r}}_{c,t}^{\text{smoothed}}$ was generated for each *c* and *t*. For each country *c*, these smoothed residuals are calculated based on weighted averages of the residuals *r*_*i*_ in the same country and other countries in the same GBD region. Weights are determined based on temporal proximity and whether or not the residual is in the country versus in the GBD region (assigning 0.99 to within‐country residuals vs. 0.01 to out‐of‐country residuals).

In the TMMP specification, the offsets *a*_*c*, *t*_ capture the smoothed residuals. Given the modelling of the U5MR on the log10 scale in the final phase, they are defined as follows:

(17) $$a_{c,t}=\log_{10}({\hat{\eta }}_{c,t}^{\text{predicted}}+{\hat{r}}_{c,t}^{\text{smoothed}})-\log_{10}({\hat{\eta }}_{c,t}^{\text{predicted}}).$$

That is, the offsets are adjustments on the log10‐scale to the values predicted by the covariate component. Given this specification of the offset and the earlier specification of the covariate term, we note that the process model for the U5MR simplifies to

$$\log_{10}\left(\eta_{c,t}\right)=\log_{10}({\hat{\eta }}_{c,t}^{\text{predicted}}+{\hat{r}}_{c,t}^{\text{smoothed}})+\epsilon_{c,t}.$$

However, in TMMP notation, we prefer to write the process model with a separate covariate component, as per Equation (15), in order to emphasise the process model's use of covariates.

#### 4.1.4 Stochastic smoothing component

The stochastic smoothing model is given by a Gaussian process, with no transformation ( B=I, so $\epsilon_{c}=\delta_{c}$)

$$\delta_{c}|\Sigma_{c}∼N\left(0,\Sigma_{c}\right),$$

where $\Sigma_{c}$ is given by the Matérn covariance function (Equation 9).

#### 4.1.5 Projection

The GBD estimation model was used to generate projections beyond the most recent observation year in each country to 2017, the end of the study period. The GBD team published long‐term projections for mortality to the year 2100 separately, using a different modelling procedure for projections (Vollset *et al*., 2020).

#### 4.1.6 Parameter estimation

For the covariate component, the coefficients are fixed to point estimates from a regression fit of the covariate component to the observed data. The hyperparameters of the stochastic smoothing component are also set to fixed values: the hyperparameters of the Matérn covariance kernel were fixed depending on a measure of data availability in each country such that areas of low data availability had higher smoothing than areas with more data. Markov chain Monte Carlo was used to sample from the joint posterior distribution of the parameters (the specific software used to do this is not provided.)

### 4.2 UN IGME model

#### 4.2.1 Process model

The process model includes systematic and stochastic smoothing components:

$$\log\left(\eta_{c,t}\right)=g_{3}(t,\eta_{c,s\neq t},\alpha_{c})+\epsilon_{c,t}.$$

The systematic component comprises a country‐specific slope and intercept:

$$g_{3}(t,\eta_{c,s\neq t},\alpha_{c})=\alpha_{c,0}+\alpha_{c,1}(t-t_{c}^{*}),$$

where $t_{c}^{*}$ refers approximately to the midpoint of the observation period. The smoothing term is defined as

$$\epsilon_{c}=B_{c}\delta_{c},$$

where $B_{c}$ contains *K*_*c*_ cubic B‐spline basis functions placed evenly over the observation period of each country (i.e. $B_{c,t,k}=b_{c,k}(t)$ where *b*_*c*, *k*_(*t*) is the *k*‐th spline basis function for country *c* evaluated at time *t*). The spline coefficients *δ*_*c*, *k*_ for $k=1,2,\ldots ,K_{c}$ follow a RW(2) process. As such, after two levels of differencing, the coefficients are normally distributed with mean zero:

(18) $$\gamma_{c,k}={△}_{2}\delta_{c,k}|\sigma_{\delta ,c}^{2}∼N(0,\sigma_{\delta ,c}^{2})$$

with

(19) $$\delta_{c,k_{c}^{*}}=0,$$

(20) $$\overset{K_{c}}{\underset{k=2}{\sum }}{△}_{1}\delta_{c,k}=0,$$

with $k_{c^{*}}$ referring approximately to the spline centred around $t_{c}^{*}$, such that $K_{0,c}=\left\{k_{c}^{*}\right\}$ and $K_{1,c}=\left\{2,\ldots ,K_{c}\right\}$. These constraints imply that $\delta_{c}$ can be recovered from the *K*_*c*_ − 2 second‐order deviations $\gamma_{c}$ by

(21) $$\delta_{c}=\left[D_{c}^{\prime }{\left(D_{c}D_{c}^{\prime }\right)}^{-1}\right]\gamma_{c},$$

where $D_{c}$ is a *K*_*c*_ × (*K*_*c*_ − 2) second‐order differencing matrix ( $D_{c,i,i}=-1,\;D_{c,i,i+1}=1$, with all other entries zero.)

#### 4.2.2 Projections

The estimates of the B3 model cover the period of observed data in each country. Projections after the last observed data point in each country can be generated by the estimation model, by projecting the second‐order differences of the process model formula. For this model, the second‐order differences are given by

$$(\log(\eta_{c,t+1})-\log(\eta_{c,t}))-(\log(\eta_{c,t})-\log(\eta_{c,t-1}))=(\epsilon_{c,t+1}-\epsilon_{c,t})-(\epsilon_{c,t}-\epsilon_{c,t-1}),$$

where the terms *ϵ*_*c*, *t*_ are obtained by extending the smoothing model to incorporate the projection period:

$$\epsilon_{c,t}=\overset{K_{c}+P_{c}}{\underset{k=1}{\sum }}b_{c,k}(t)\delta_{c,k},$$

where *b*_*c* *k*_(*t*) is the *k*‐th spline function in country *c* evaluated at time *t* and *P*_*c*_ refers to the number of spline functions added. The default projections implied by the estimation model would project forward the RW(2) stochastic smoothing component defined above. However, the authors found that this approach can lead to unrealistically high or low projections of U5MR for countries with longer term projections. As such, they used a logarithmic pooling approach to adjust the projections, combining country‐specific projections with information on global rates of change. The coefficients *δ*_*c*, *k*_ are extrapolated as follows:

$$\begin{array}{cc} \delta_{c,k+1} & \;=2\delta_{c,k}-\delta_{c,k-1}+\gamma_{c,k},\\ \gamma_{c,k}|\Gamma_{c,k},\Theta_{c,k} & \;∼N\left(\Gamma_{c,k},\Theta_{c,k}\right), \end{array}$$

where

$$\begin{array}{cc} \Gamma_{c,k} & =W\cdot G+(1-W)\cdot {△}_{2}\delta_{c,k-1},\\ \Theta_{c,k} & =W\cdot V+(1-W)\cdot \Theta_{c,k-1}, \end{array}$$

with *G* and *V* equal to the median and variance of the estimates of past second‐order differences ${△}_{2}{\hat{\delta }}_{1:C,1:K_{c}}$'s respectively, and $\Theta_{c,K_{c}-1}=\sigma_{\delta ,c}^{2}$. The parameter 0 ≤ *W* ≤ 1 controls the pooling weight.

#### 4.2.3 Parameter estimation

The variances $\sigma_{\delta ,c}^{2}$ of the random walk deviations *δ*_*c*, *k*_ are estimated hierarchically so that information on the degree of smoothing can be shared between countries. Conversely, the parameters *α*_*c*, 0_ and *α*_*c*, 1_ controlling the intercept and slope of the systematic component are estimated independently with vague priors. The pooling weight parameter *W* was fixed to a value chosen through an out‐of‐sample validation exercise. Bayesian inference was performed using Markov chain Monte Carlo, implemented with the software JAGS (Plummer, 2003).

### 4.3 Comparing the Under‐Five Mortality Rate Models

A full accounting for why the estimates from the GBD and UN IGME models differ requires consideration of differences in data sources, data preprocessing and the data models in addition to differences in their process models. Casting the models in TMMP notation, however, provides an organising framework for doing so. With both models expressed using the TMMPs notation, it is easier to list the main assumptions made in a standardised way and compare assumptions across models.

Table 5 summarises the process model and parameter estimation strategies of each model. Both models use a logarithmic transformation of *η*, with logarithm base 10 used by the GBD model as compared with a natural log‐transform used by UN IGME. Based on using a log‐transformation, neither model imposes an upper bound to U5MR—presumably not needed given that U5MR outcomes are typically well below 0.5—and both models capture differences in U5MR on a relative scale. The difference between log10 and natural log‐transforms results in a difference in interpretation of the scale of the relative differences in the components of the process model.

Comparing components other than the stochastic smoothing component, the GBD model uses several covariates (LDI, EDU and HIV), and an offset estimated in a separate regression, as compared with the use of a linear systematic trend that is non‐zero during the observation period in the B3 model. The two models use different smoothing approaches, with the GBD model using a Gaussian process and the B3 model using a second‐order random walk on B‐spline coefficients. Finally, the GBD model fixes model parameters in earlier steps in its estimation procedure, while B3 uses full Bayesian inference to estimate parameter uncertainty during the observation period. B3 uses hierarchical distributions to share information between countries on the variability of the smoothing term only. For the GBD model, the offsets estimated in a separate step by smoothing residuals across time and space serve as a way of sharing information across countries.

Using this comparison, we can interpret how the models will behave in forward projections. The GBD model's projections are informed by its covariate model and systematic offsets. Given its usage of a stationary smoothing model, deviations away from these components in recent years will converge back to zero; hence, the estimates of U5MR will convergence back to the covariate‐plus‐offset‐based estimates. The B3 model short‐term projections, on the other hand, derive entirely from the stochastic smoothing component, extrapolating recently observed linear trends (in log space) into the future, while rates of change in longer term projections converge to a global distribution of past observed rates of change.

## 5 Additional Examples

In this section, we describe the process models of four existing models for different health indicators.

### 5.1 Family Planning Estimation Model

The Family Planning Estimation Model (FPEM) is a country‐level model of contraceptive use rates among married women of reproductive age from 1990 to 2020 (Cahill *et al*., 2018) and is an updated version of an earlier model (Alkema *et al*., 2013). The full model breaks down the total use rate into traditional and modern contraceptive method users and models in addition the unmet need for contraceptives. For the purposes of this paper, we will only examine how it models the total contraceptive use rate and refer readers to the paper for additional details.

#### 5.1.1 Indicator of interest

Let *η*_*c*, *t*_ be the population mean total contraceptive use among married or in‐union women age 15–49 in country *c* in year *t*, where total contraceptive use is defined as the proportion of women who report using at least one contraceptive method of any type.

#### 5.1.2 Process model

The process model includes a systematic and smoother component:

$$\text{logit}(\eta_{c,t})=g_{3}(t,\eta_{c,s\neq t},\alpha_{c})+\epsilon_{c,t}.$$

**FIGURE 3:**
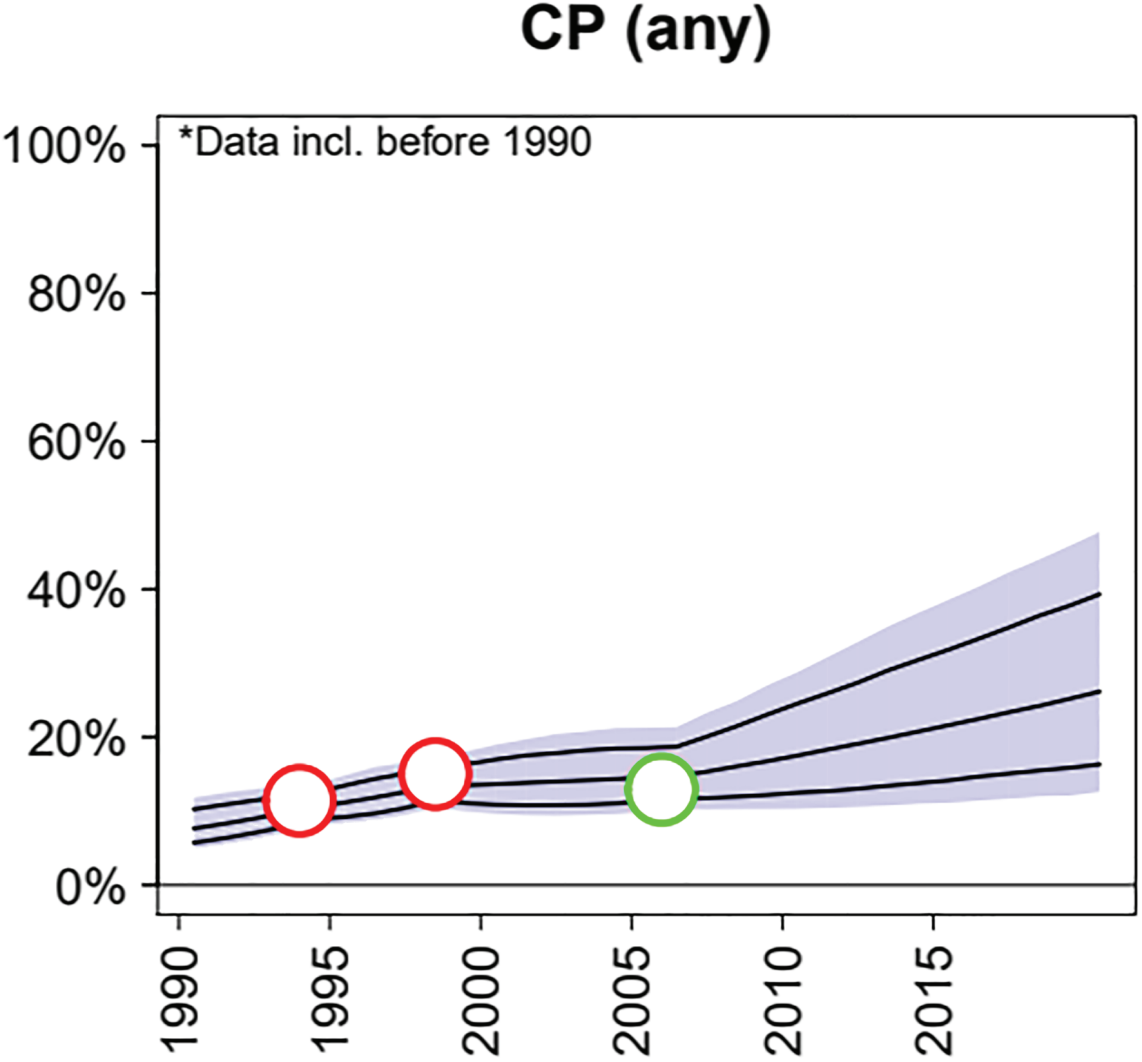
Family planning estimation model (FPEM, Alkema *et al*., 2013) estimates for Côte d'Ivoire. The systematic component of the model informs forward projections and estimates in countries with limited data available. Copyright CC BY 4.0 [Colour figure can be viewed at wileyonlinelibrary.com]

Adoption of contraception at the country level is expected to start slowly, speed up and then slow down before reaching an asymptote. The model incorporates this expectation of an S‐shaped trend through the systematic component. The systematic component encodes the rate of change of a logistic curve. A reference year *t* ^∗^ is fixed, and the systematic trend is propagated forward and backward from the reference year. At *t* ^∗^, we set $g_{3}(t^{*},\eta_{c,s\neq t},\alpha_{c})=\Omega_{c}$, where Ω_*c*_ is the level in the reference year. When *t* > *t* ^∗^, the systematic component propagates forward:

$$\begin{array}{cc} g_{3}(t,\eta_{c,s\neq t},\alpha_{c})|t>t^{*} & =\text{logit}\left(\eta_{c,t-1}\right)\;+\iota_{c,t},\\ & =\left\{\begin{array}{cc} \text{logit}\left({\tilde{P}}_{c}\cdot {\text{logit}}^{-1}\left(\text{logit}\left(\frac{\eta_{c,t-1}}{{\tilde{P}}_{c}}\right)+\omega_{c}\right)\right),\; & \;\text{when}\;\eta_{c,t-1}<{\tilde{P}}_{c}\\ \;\text{logit}\left(\eta_{c,t-1}\right),\; & \;\text{otherwise,} \end{array}\right. \end{array}$$

where ${\tilde{P}}_{c}$ is an asymptote, *ω*_*c*_ a rate parameter and $\alpha_{c}=\left\{\tilde{P},\omega_{c},\Omega_{c}\right\}$. A similar equation is applied when *t* < *t* ^∗^ to extend the systematic trend backwards from the reference year. This component encodes the assumption that contraceptive adoption in each country follows the same shape but may differ in its timing, rate and peak adoption. The effect of the systematic component can be seen in the model's projections and fits in data‐sparse countries. In Côte d'Ivoire, the model projects an increase in contraceptive use because the country's systematic trend, with hyperparameters informed by other countries in the region, puts the country on the verge of its contraceptive transition (Figure 3).

Deviations from the expected rate of change given by the systematic component are captured via an AR(1) stochastic smoothing component. Using the notation of our proposed model class, this can be expressed as placing a multivariate normal distribution on the vector of deviations:

$$\delta_{c}|\Sigma_{c}∼N(0,\Sigma_{c}),$$

where $\Sigma_{c}$ is given by the AR(1) autocovariance function, and $\epsilon_{c}=\delta_{c}$. These deviations are added to the systematic component, which for time *t* > *t* ^∗^ depends on *η*_*c*, *t* − 1_. As such, the smoothing terms *ϵ*_*c*, *t*_ represent deviations in the rate of change of modern contraceptive use within a country. Because these smoothing terms accumulate over time, variance increases with the projection horizon.

#### 5.1.3 Parameter estimation

The FPEM introduces many country‐level parameters that need to be estimated. The systematic component for FPEM has parameters for the asymptote, rate and timing of each country's logistic curve that describes its adoption of contraceptives. Hierarchical distributions share information about each parameter between countries. First, a transformation was applied to constrain the asymptote ${\tilde{P}}_{c}$ to be between 0.5 and 1, given an unconstrained parameter ${\tilde{P}}_{c}^{*}$. The unconstrained asymptote parameters have one level of hierarchy:

$${\tilde{P}}_{c}^{*}|{\tilde{P}}_{w}^{*},\sigma_{{\tilde{P}}^{*}}^{2}∼N\left({\tilde{P}}_{w}^{*},\sigma_{{\tilde{P}}^{*}}^{2}\right),$$

where ${\tilde{P}}_{w}^{*}$ is the world mean asymptote and $\sigma_{\tilde{P}*}^{2}$ describes the variation around that mean. The rate parameters have three levels of hierarchy, in that they are nested first within subregions and regions. First, a transformation was applied to constrain the rate parameter *ω*_*c*_ to be between 0.01 and 0.5, given an unconstrained parameter $\omega_{c}^{*}$. Then, a three‐level hierarchical model is used for the unconstrained parameter $\omega_{c}^{*}$:

$$\begin{array}{cc} \omega_{c}^{*}|\omega_{s[c]}^{*},\sigma_{\omega_{s}^{*}}^{2}\; & ∼N(\omega_{s[c]}^{*},\sigma_{\omega_{s}^{*}}^{2}),\\ \omega_{s}^{*}|\omega_{r[s]}^{*},\sigma_{\omega_{r}^{*}\;}^{2} & \;∼N(\omega_{r[s]}^{*},\sigma_{\omega_{r}^{*}}^{2}),\\ \omega_{r}^{*}|\omega_{w}^{*},\sigma_{\omega_{w}^{*}\;}^{2} & \;∼N(\omega_{w}^{*},\sigma_{\omega_{w}^{*}}^{2}), \end{array}$$

where *s*[*c*] indexes the subregion of country *c* and *r*[*s*] indexes the region of subregion *s*. The hierarchical structure for the timing parameters Ω_*c*_ depends on whether the country is classified as developing or developed. For developing countries, three levels of hierarchy are used (subregion, region and world); for developed countries, only one level is used. The difference in hierarchical structure between $\tilde{P}$ and Ω_*c*_,  *ω*_*c*_ arises from substantive knowledge, in that rate is expected to differ regionally, and the timing may vary for developing countries, while the asymptote is expected to be comparable across regions.

### 5.2 Neonatal Mortality Rate Model

Alexander & Alkema (2018) model the NMR in 195 countries from at latest 1990 to 2015, and was adopted by the UN IGME. The model set up incorporates the strong relationship between NMR and U5MR: as U5MR increases, the ratio of neonatal to other child mortality tends to decrease. As such, this model provides an example of how covariates can be used in our proposed model class.

#### 5.2.1 Indicator of interest

Rather than model the NMR directly, the authors chose to model the ratio of the neonatal mortality rate to the rate of non‐neonatal deaths. This was motivated by the strong relationship between the ratio and U5MR and also to constrain the NMR to be smaller than the U5MR. As such, the indicator of interest is defined as *η*_*c*, *t*_ = $\eta_{c,t}^{\left(neo\right)}/(X_{c,t}-\eta_{c,t}^{\left(neo\right)})$, where $\eta_{c,t}^{\left(neo\right)}$ and *X*_*c*, *t*_ are the NMR and U5MR in country *c* at time *t*.

#### 5.2.2 Process model

**FIGURE 4:**
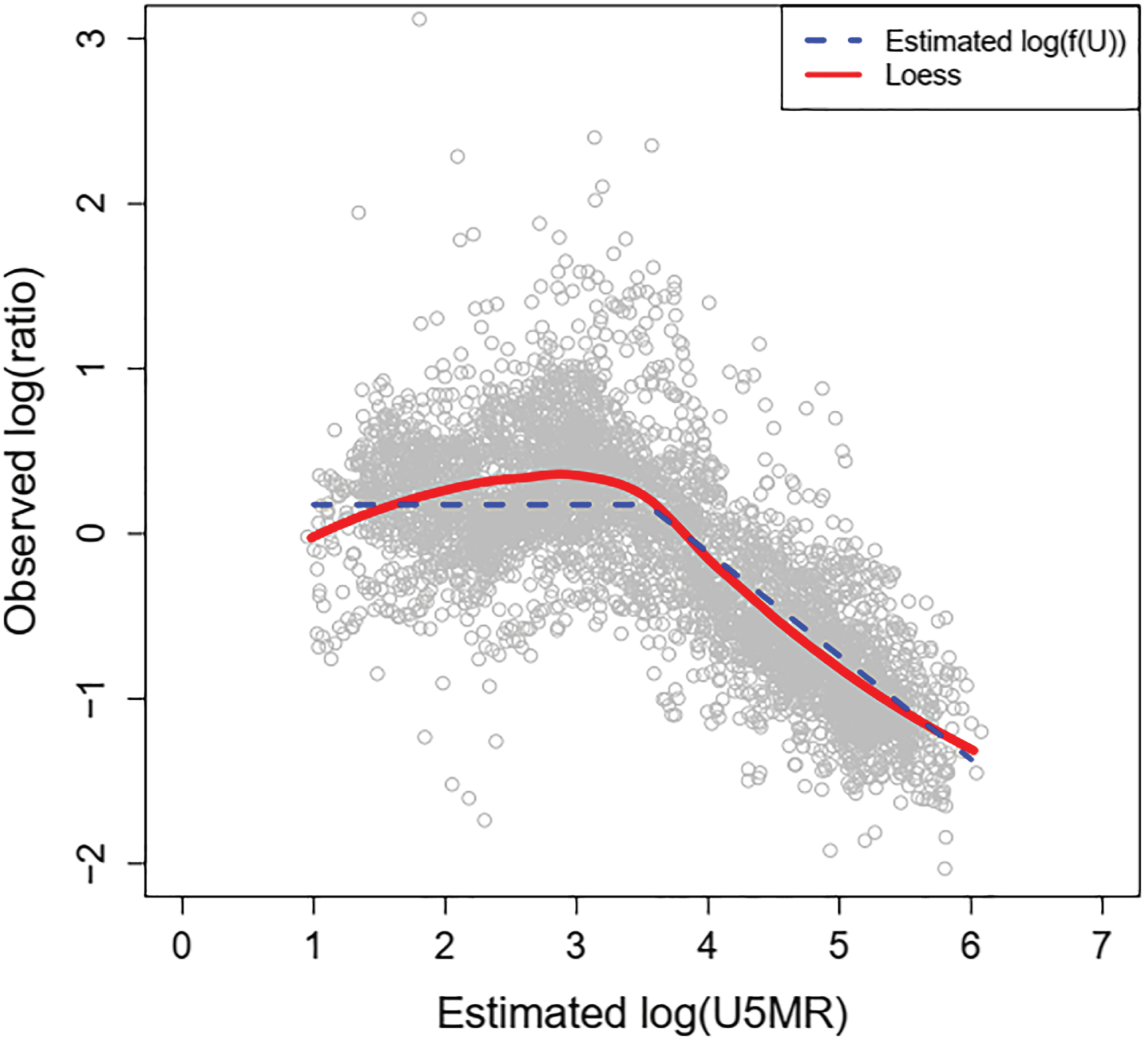
The relationship between log(U5MR) and the ratio of neonatal deaths to non‐neonatal deaths observed in the dataset used for the neonatal mortality rate model of Alexander et al. (2018). Copyright CC BY 3.0 DE [Colour figure can be viewed at wileyonlinelibrary.com]

The process model includes a covariate and stochastic smoothing component:

$$\log(\eta_{c,t})=g_{2}(X_{c,t},\beta_{c})+\epsilon_{c,t},$$

where, as before, *X*_*c*, *t*_ is the U5MR for country *c* at time *t*. The covariates do not vary over time, so we write them as a vector $\beta_{c}$ for each country. The authors found through exploratory data analyses that the relationship between U5MR and the log ratio is constant up to a cut‐off U5MR value and then decreases linearly (Figure 4). As such, they chose a piecewise linear form for the function *g*_2_:

$$g_{2}(X_{c,t},\beta_{c})=\beta_{c,0}+\beta_{1}\cdot \left(\log(X_{c,t})-\log(\beta_{2})\right)1_{\left[X_{c,t}>\beta_{2}\right]},$$

where *β*_*c*, 0_ and *β*_1_ are intercept and slope parameters, and *β*_2_ is the cut‐off U5MR value at which point the relationship becomes constant.

The deviations *ϵ*_*c*, *t*_ capture trends in the data not explained by the covariate component and are modelled with B‐splines, similar to the B3 model. In TMMP class notation, we can write

$$\epsilon_{c}=B_{c}\delta_{c},$$

where $B_{c}$ contains *K*_*c*_ cubic B‐spline basis functions, placed at evenly spaced knot locations. Sufficient knots are placed in each country to cover the period for which data are available. The spline coefficients $\delta_{c}$ are modelled with an RW(1) process, which after one level of differencing is multivariate normally distributed with mean zero. That is,

(22) $$\gamma_{c,k}=△\delta_{c,k}|\sigma_{\gamma ,c}^{2}∼N(0,\sigma_{\gamma ,c}^{2}),$$

with

(23) $$\overset{K_{c}}{\underset{k=1}{\sum }}\delta_{c,k}=0,$$

such that $K_{0,c}=\left\{1,\ldots ,K_{c}\right\}$. Note that the sum‐to‐zero constraint implies that $\delta_{c}$ can be recovered from the *K*_*c*_ − 1 deviations $\gamma_{c}$ by

(24) $$\delta_{c}=\left[D_{c}^{\prime }{\left(D_{c}D_{c}^{\prime }\right)}^{-1}\right]\gamma_{c},$$

where D is a *K*_*c*_ × (*K*_*c*_ − 1) first‐order differencing matrix (i.e. $D_{i,i}=-1,\;D_{i,i+1}=1$, and is zero everywhere else.)

**FIGURE 5:**
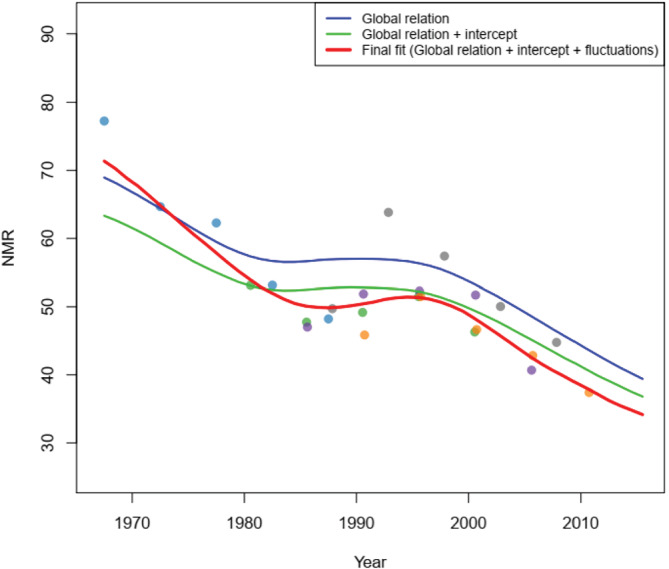
Example estimates for neonatal mortality from Alexander and Alkema (2018). Global relation (blue) shows the estimates from the covariate component of the process model, global relation + intercept (green) the covariate estimates plus a country‐specific intercept, and final fit (red) shows the final estimates that include the smoothing model. The smoothing model has the most impact within the data support, as it modifies the expected trend based on covariates to better fit the observed data. Copyright: CC BY 3.0 DE [Colour figure can be viewed at wileyonlinelibrary.com]

Figure 5 shows model estimates separated into covariate and stochastic smoothing components, which illustrates how projections are influenced by the relationship between U5MR and covariates. The projection follows the expected trend based on covariates closely, with a country‐specific offset. The stochastic smoothing component has a larger effect on the model fit where there are data available, showing how the model modifies the expected trend from covariates to fit the observed NMR data.

#### 5.2.3 Projections

The main model produces estimates that cover the period of observed data in each country. As per the TMMP specification, for the NMR model with the non‐stationary RW(1) smoother with r=1, projections for the period after the last observed data point in each country to 2015 are obtained by projecting first‐order differences that follow from the process model expression. Here, the first‐order differences are given by

(25) $$\eta_{c,t+1}-\eta_{c,t}=\beta_{1}\cdot \left(\log(X_{c,t+1})1_{\left[X_{c,t+1}>\beta_{3}\right]}-\log(X_{c,t})1_{\left[X_{c,t}>\beta_{3}\right]}\right)+\epsilon_{c,t+1}-\epsilon_{c,t},$$

where the smoothing terms *ϵ*_*c*, *t*_ are obtained from extending the spline smoothing model

(26) $$\epsilon_{c,t}=\overset{K_{c}+P_{c}}{\underset{k=1}{\sum }}b_{c,k}(t)\delta_{c,k},$$

where *P*_*c*_ refers to the total number of spline functions added and the *δ*_*c*, *k*_ are projected based on the RW(1) process:

(27) $$\delta_{c,k}=\delta_{c,k-1}+\gamma_{c,k},$$

(28) $$\gamma_{c,k}|\sigma_{\gamma ,c}^{2}∼N(0,\sigma_{\gamma ,c}^{2}).$$

#### 5.2.4 Parameter estimation

The smoothing model estimates some country‐level parameters independently and others hierarchically, which encodes assumptions about how neonatal mortality rates are related across countries. The intercepts *β*_*c*, 0_ were estimated hierarchically, as were the variances of the smoothing model deviations. All other hyperparameters were assigned vague priors.

### 5.3 Bayesian Maternal Mortality Model

The Bayesian maternal mortality model, referred to as ‘Bmat’, is used to estimate the maternal mortality ratio (maternal deaths per 100 000 live births) in countries from 1985 to 2017 (Alkema *et al*., 2017; United Nations Maternal Mortality Estimation Inter‐Agency Group (MMEIG), 2015; 2019). The model is an extension of a previous multilevel regression modelling approach (Wilmoth *et al*., 2012) used by the United Nations Maternal Mortality Estimation Inter‐agency group (UN MMEIG). Bmat incorporates covariates similarly to the earlier model, while also allowing data‐driven deviations from the expected covariate trend. The Bmat model handles AIDS and non‐AIDS maternal deaths separately; for the purposes of this paper, we will focus on the model of non‐AIDS maternal deaths.

#### 5.3.1 Indicator of interest

Let *η*_*c*, *t*_ represent the population proportion of non‐AIDS deaths to women of reproductive age that are of a maternal cause in country *c* in year *t*.

#### 5.3.2 Process model

Bmat models the expected trend in *η* by a combination of covariate and smoothing model components:

$$\log\left(\eta_{c,t}\right)=g_{2}(X_{c,t},\beta_{c})+\epsilon_{c,t},$$

where $X_{c,t}$ is a set of covariates for country *c* at time *t*, and $\beta_{c}$ are associated regression coefficients (the regression coefficients do not vary over time, yielding a vector of coefficients $\beta_{c}$ for each country.)

The covariate model is based on the earlier UN MMEIG regression model and is given by

$$g_{2}(X_{c,t},\beta_{c})=\beta_{c,0}+\beta_{1}\log(X_{c,t}^{GDP})+\beta_{2}\log(X_{c,t}^{GFR})+\beta_{3}X_{c,t}^{SAB}.$$

The deviations are smoothed with an ARIMA(1, 1, 1) process, which is a stationary autoregressive moving average (ARMA) process after differencing once ( r=1). In TMMP notation

$$\begin{array}{cc} \epsilon_{c} & \;=\delta_{c},\\ \delta_{c,t} & \;=0,\text{for}\;t=1990,\\ {△}_{1}\delta_{c}|\Sigma_{c} & \;∼N(0,\Sigma_{c}), \end{array}$$

where the covariance matrix $\Sigma_{c}$ is specified via an autocovariance function *s* that captures the autocovariance of an ARMA(1, 1) process, parametrised with country‐specific stationary variance $\sigma_{\delta ,c}^{2}$, autoregressive parameter 0 ≤ *ρ* ≤ 1 and moving average parameter −1 ≤ *θ* ≤ 0.

**FIGURE 6:**
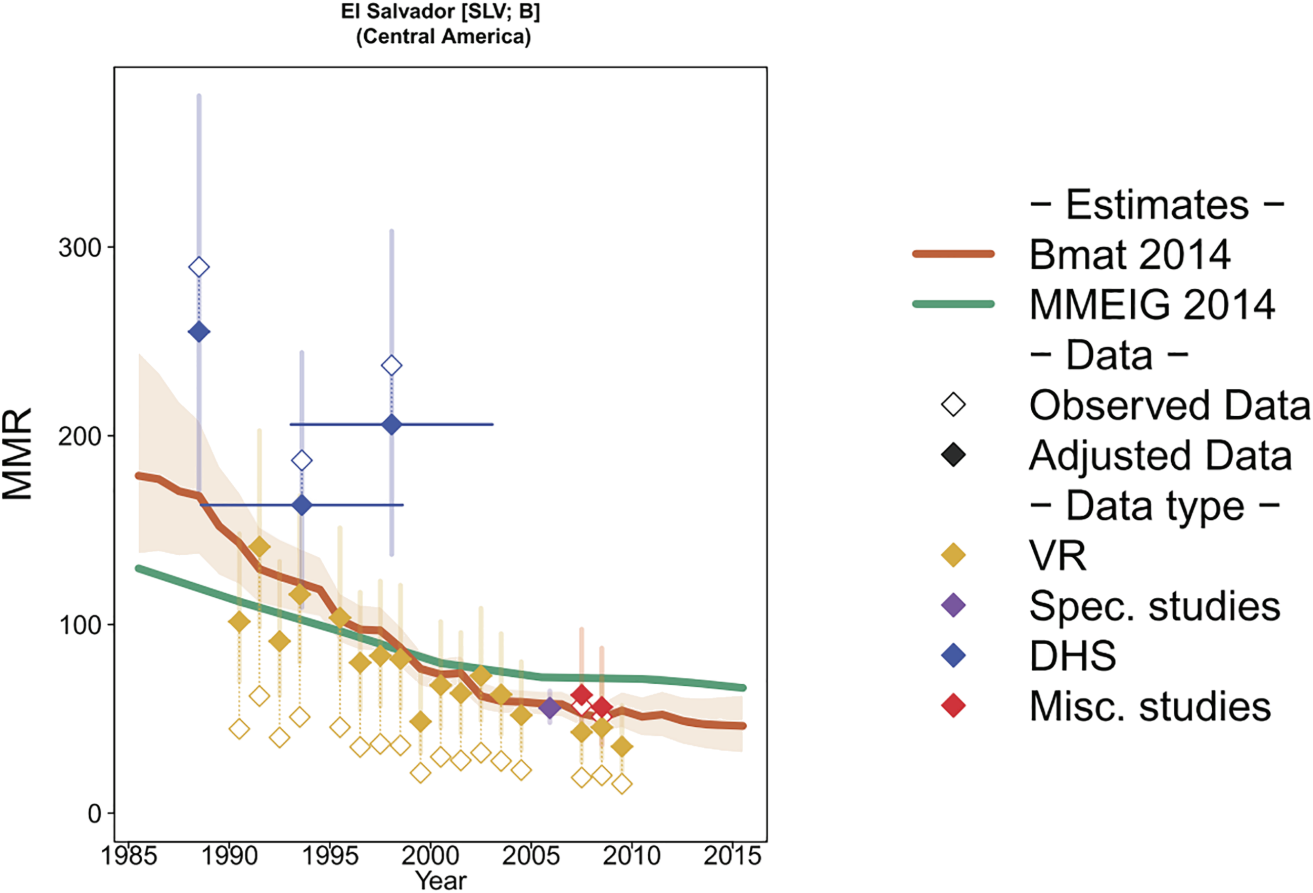
Bmat estimates of maternal mortality for El Salvador. Bmat estimates are displayed in red and follow bias‐adjusted data more closely than covariate‐driven estimates based on the UN MMEIG 2014 model. Reproduced with permission [Colour figure can be viewed at wileyonlinelibrary.com]

The addition of a smoothing model increases the flexibility of the process model. Model estimates combine covariate trends and patterns in the observed maternal mortality data, as illustrated in Figure 6 for El Salvador: the model‐based estimates deviate from the expected covariate‐based trend and better fits bias‐adjusted historical DHS data and high‐quality VR data, when available.

#### 5.3.3 Parameter estimation

The intercepts *β*_*c*, 0_ are estimated hierarchically with two levels (country within region within world). The other regression coefficients are shared between all countries and are given a vague prior. The country‐specific variance parameter of the ARIMA(1, 1) smoothing model is estimated hierarchically in order to share information between countries:

$$\sigma_{\delta ,c}=\sigma_{\delta ,w}\cdot (1+\lambda_{c})\;\lambda_{c}|\sigma_{\lambda }^{2}∼TN_{\left(-1,2\right)}(0,\sigma_{\lambda }^{2}).$$

The parameter *σ*_*δ*, *w*_ represents a central value of the variances across all countries, and *λ*_*c*_ is a country‐specific multiplier. A truncated normal prior is placed on *λ*_*c*_, limiting its values to fall between −1 and 2. This hierarchical structure allows different countries to have differing levels of smoothness in their fluctuations, while still sharing information between countries.

### 5.4 Bayesian Model for Subnational Mortality

Alexander *et al*. (2017) developed a model for estimating age‐specific mortality rates at the subnational level, focusing on obtaining estimates by county in the United States. Unlike the other examples mentioned in this paper, this model set up is more of a ‘traditional’ demographic model as it deals explicitly with modelling age patterns in mortality over time, rather than focusing on modelling temporal trends in an aggregate indicator of mortality. The challenge with estimating mortality age schedules at the subnational level is that, when populations are small, stochastic variation is high, and so the underlying mortality risk curve may be unclear or uncertain from the observed data.

In essence, Alexander *et al*. (2017) built on the traditional demographic method of using model age schedules of mortality, but placed this within a Bayesian framework which allows for stochastic estimates and forecasts and increased flexibility in estimates. We include it as an example here to show that both demographic models and models for global health indicators can be expressed in the same generalised TMMP framework.

#### 5.4.1 Indicator of interest

The indicator *η*_*a*, *c*, *t*_ is defined as the population mortality rate for age *a* in county *c* at time *t*.

#### 5.4.2 Process model

The process model incorporates a covariate and stochastic smoothing component:

$$\log\left(\eta_{a,c,t}\right)=g_{2}(X_{a},\beta_{c,t})+\epsilon_{a,c,t},$$

where $X_{a}=\left\{X_{a,1},X_{a,2},X_{a,3}\right\}$ are the first three principal components of a set of standard mortality curves and $\beta_{c,t}$ is the associated regression coefficients that differ by county and time point. The regression is equation is given by

$$g_{2}\left(X_{a},\beta_{c,t}\right)=\beta_{c,t,1}\cdot X_{a,1}+\beta_{c,t,2}\cdot X_{a,2}+\beta_{c,t,3}\cdot X_{a,3}.$$

Whereas previous model examples with covariates have focused on relations between country‐level indicators, the covariates here capture the main sources of variation in mortality over age, which allows for plausible estimates over age‐specific mortality to be imputed, and also ‘smooths out’ noisy age‐specific mortality curves that are commonly observed in small populations.

The *ϵ*_*a*, *c*, *t*_ term was included in the model to account for deviations away from standard patterns commonly seen in mortality rates. Unlike previous examples, no temporal model is placed on the deviations *ϵ*_*a*, *c*, *t*_. Instead, the deviations are shrunk towards zero within each age group:

$$\epsilon_{a,c,t}|\sigma_{a}^{2}∼N(0,\sigma_{a}^{2}),$$

where $\sigma_{a}^{2}$ is the variance for age group *a*.

#### 5.4.3 Parameter estimation

The process model requires estimating regression coefficients *β*_*c*, *t*, *p*_ for every county, time point and principal component. To share information across counties, a hierarchical prior is placed on the regression coefficients:

$$\beta_{p,c,t}|\mu_{p,t},\sigma_{p,t}^{2}∼N\left(\mu_{p,t},\sigma_{p,t}^{2}\right).$$

Further structure is placed on the hyperparameters *μ*_*p*, *t*_ in order to smooth over time, with the assumption being that the underlying expected trend in each dimension is smooth:

$$\mu_{p,t}|\mu_{p,t-1},\mu_{p,t-2},\sigma_{\mu }^{2}∼N(2\mu_{p,t-1}-\mu_{p,t-2},\sigma_{\mu }^{2}).$$

Non‐informative priors were set on the remaining process model hyperparameters.

## 6 Discussion

In this paper, we have introduced a general model class, ‘TMMPs’, which encompasses many existing demographic and health models. The key structural feature of the class is that it makes a distinction between the data and process model, separating the dynamics of an indicator from details of how the observed data were generated. Thus, the process model expresses the modeler's assumptions about the dynamics driving the latent value of the indicator, and the data model describes assumptions about noise and error in the observed data. Once a model is shown to fall into this model class, it is easier to compare the assumptions made in different models.

We showed how six existing models of demographic and health indicators fit into the model class. The example models were for a diverse set of outcomes: under‐five mortality, total contraceptive use, neonatal mortality, maternal mortality and subnational mortality rates. These models incorporated a variety of modelling approaches, including Gaussian process regression, ARIMA time series, and penalised spline regressions. The GBD Study U5MR model in particular is the most different of the example models based on how it was originally presented as a three‐stage modelling procedure (Dicker *et al*., 2018). We chose to focus on how the final set of estimates was produced from the GBD model, hence casting their third modelling stage as a TMMP model. The first two modelling stages were accommodated through the use of an offset component in the process model. As such, the multistage approach resulted in using the same data twice at the final stage (once for defining the fixed data‐driven offset in the process model for *η*, and once in the TMMP data model). To more fully describe models with multiple stages such as the GBD model, we recommend writing each stage as its own TMMP, with expanded notation to account for how data are used and reused through the modelling stages. In case of the GBD modelling approach, this would help make exact how the offset term was obtained.

We recommend that the writing of model assumptions for TMMPs in a standardised way is considered for a future version of GATHER, referring to Guidelines for Accurate and Transparent Health Estimates Reporting (Stevens *et al*., 2016). We provided a template for doing so in Appendix S3. Generally, we expect that it will be possible to express a complete model in the TMMP class within the provided template. The overall goal, however, is to facilitate communicating important modelling assumptions. Hence, for more complex models, authors can consider filling out multiple templates, with one template serving as the main one for communicating the main assumptions while others may focus on additional details related to the process model structure or parameter estimation.

The models we included as examples in this paper are by no means an exhaustive list of the models than can be described within our framework. Many spatial models, which directly model spatial correlation between units, can be expressed in our class. Spatial smoothing models following the work of Knorr‐Held (2000) in particular are an example of a modelling approach that falls into our model class. Additional research is needed to investigate how other modelling approaches relate to TMMPs. The structure of the TMMP model class is flexible and extendable, making it possible to propose new components as necessary to capture models currently outside of the class.

So far, we have focused on describing the model class and showing how existing models fall into it, rather than evaluating the assumptions made by each model. In the future work, we plan to compare in more detail the effects of the particular choice for each component on the resulting estimates. A better understanding of the effects of various smoothing models, for example, is helpful both for analysing the behaviour of existing models and for developing new models.

The number of models developed for providing demographic and health indicator estimates has perhaps grown faster than tools to interpret their results and how they relate to one another. The model class proposed in this paper is one tool that can be used to understand model assumptions. We hope it will facilitate interpreting, comparing and contrasting existing models that fall within the model class as well as the development of new modelling approaches.

## Supporting information

INSR_12491_supplemental.pdf
