# Supplementary material for: Temporal Models for Demographic and Global Health Outcomes in Multiple Populations: Introducing a New Framework to Review and Standardise Documentation of Model Assumptions and Facilitate Model Comparison
Source: Int Stat Rev. 2022 Mar 7;90(3):437–67. doi: 10.1111/insr.12491 (PMC9790657; doi:10.1111/insr.12491)
Supplement: Supplementary file 1 — INSR_12491_supplemental.pdf [file INSR-90-437-s001.pdf]

Appendix S1

In this appendix we describe both models from the U5MR case study as they were originally presented, rather than in TMMP notation.

Global Burden of Disease (GBD) Model

The GBD model (Dicker et al., 2018) has three modeling stages. The starting point is the observed U5MR data, denoted y_i, $i = 1, \ldots, N$, which are derived from vital registration systems, sample vital registration systems, surveys, and censuses. Estimates of the stochastic error and non-sampling error associated with each observation were computed by different methods according to the data source type; for example, for observations from complete vital registration systems, the stochastic error is computed according to a binomial model and the non-sampling error is assumed to be zero.

The goal of this description is to present the parts of the GBD modeling procedure relevant to the TMMP process model; interested readers should refer to Supplemental Materials 1 of Dicker et al. (2018) for more details on the data sources used and the data preprocessing performed.

First Stage The first stage of modeling does an initial adjustment of the observed data, to account for biases from each data source. A nonlinear mixed effects regression model is fit to the U5MR observations, using lag-distributed income per capita (LDI), mean years of education for women of reproductive age (15-49 years), and HIV death rate in ages 0-4, as covariates:

$$y_i = \exp\left[(\beta_1 + \gamma_{1,c[i]})\log(x^{\text{LDI}}_{c[i],t[i]}) + (\beta_2 + \gamma_{2,c[i]})x^{\text{educ}}_{c[i],t[i]} + \gamma_{c[i]} + \gamma_{c[i],s[i]} + \alpha_{type[i]}\right] + (\beta_3 + \gamma_{3,c[i]})x^{\text{HIV}}_{c[i],t[i]} + \epsilon_{c[i],t[i],s[i]}, \quad (1)$$

where

- $c[i]$, $t[i]$, $s[i]$, and $type[i]$ are the country, year, data source, and data source type of observation i, respectively.
- $\beta_1, \beta_2, \beta_3$, and $\alpha_{type[i]}$ are fixed effects.
- $\gamma_{c[i]}$, $\gamma_{1,c[i]}$, $\gamma_{2,c[i]}$, $\gamma_{3,c[i]}$ and $\gamma_{c[i],s[i]}$ are random effects. The description of the model does not specify the distribution of the random effects. (In addition, the equation supplied by the authors on p.15 does not include a random intercept $\gamma_{3,c[i]}$ for the HIV covariate but this term is included in the related equation on p.16; we did not receive clarification from the corresponding author, so we took the omission on p.15 to be an error and included the random effect.)
- ϵ_i refers to the measurement error of observation i. The description of the model does not specify the distribution of the measurement errors.

Note that the regression model given above does not necessarily respect the bounds of the observed data (which are in $[0, 1000]$) and may result in U5MR predictions that are not well defined.

We denote the fitted coefficients and random effects from the above model as $\hat{\beta}_1$, $\hat{\beta}_2$, and so forth. Bias adjusted data points $\hat{y}_i^{\text{adjusted}}$ were derived from the fitted coefficients and mixed effects according to the following formula:

$$\hat{y}_i^{\text{adjusted}} = \exp\left[(\hat{\beta}_1 + \hat{\gamma}_{1,c[i]})\log(x^{\text{LDI}}_{c[i],t[i]}) + (\hat{\beta}_2 + \hat{\gamma}_{2,c[i]})x^{\text{educ}}_{c[i],t[i]} + \hat{\gamma}_{c[i]} + \hat{\gamma}_{c[i],ref} + \hat{\alpha}_{ref,c[i]}\right] + (\hat{\beta}_3 + \hat{\gamma}_{3,c[i]})x^{\text{HIV}}_{c[i],t[i]} + \hat{\epsilon}_{c[i],t[i],s[i]}, \quad (2)$$

where $\hat{\gamma}_{ref,c[i]}$ and $\hat{\alpha}_{ref,c[i]}$ are the random effects from one or more reference data sources, chosen on a country by country basis, that are deemed to be more reliable than other sources by the study team.

The model was then used to generate predictions for the U5MR in every country and time point, without random effects or survey type fixed effects:

$$\hat{\eta}^{\text{predicted}}_{c,t} = \exp\left(\hat{\beta}_1 \log(x^{\text{LDI}}_{c,t}) + \hat{\beta}_2 x^{\text{educ}}_{c,t} + \hat{\alpha}\right) + \hat{\beta}_3 x^{\text{HIV}}_{c,t}. \quad (3)$$

We interpret the intercept $\hat{\alpha}$ as being derived from the α_{ref} term in the regression equation, but the exact definition is not made clear in the authors' description. We call this predicted time series $\hat{\eta}_{c,t}^{\text{predicted}}$ because they are predictions of the true U5MR in every country and time point.

Finally, let $r_i = \hat{y}_i^{\text{adjusted}} - \hat{\eta}_{c[i],t[i]}^{\text{predicted}}$ be the residuals between the predicted time series and the adjusted observations. The next step smooths these residuals over time and space, and then adds the smoothed residuals back to the predicted time series to yield a new set of predictions.

Second Stage: spatiotemporal smoothing of the residuals The goal is to obtain $\hat{r}_{c,t}^{\text{smoothed}}$, a smoothed version of the residuals $\hat{r}_i$ for each country c and year t. Note that r_i is defined in terms of observations (each observation has a residual), but that $\hat{r}_{c,t}^{\text{smoothed}}$ is defined in terms of country-years (every country-year is assigned one smoothed residual value.) The values for $\hat{r}_{c,t}^{\text{smoothed}}$ are obtained from the residuals r_i within the same country (referred to as in-country residuals) and from residuals in countries within the same GBD region (out-of-country residuals) through a weighting approach. Weights are determined by spatial and temporal proximity, varying uncertainty associated with the r_i's is not taken into account.

Regarding the combining of in-country and out-of-country residuals, the supplemental material states that "we gave 99% of the weight to in-country residuals, and 1% of the weight to out-of-country residuals" (p. 17). No equation was given. We interpret this statement to mean that for country of interest c^*, spatial proximity weights are given by $w_{i,c^*}^{(space)} = 0.99$ for i with $c[i] = c^*$, $w_{i,c^*}^{(space)} = 0.01$ for i with $c[i] \neq c^*$ but in the same GBD region, and zero otherwise, i.e.

$$w_{i,c^*}^{(space)} = 0.99 \cdot \mathbb{I}(c[i] = c^*) + 0.01 \cdot \mathbb{I}(c[i] \neq c^*, c[i] \in GBD(c^*)), \tag{4}$$

where $\mathbb{I}$ is the indicator function

Residuals were also weighted based on temporal proximity. For year of interest t^* and country of interest c^*, we denote the weight to account for temporal proximity by $w_{i,c^*,t^*}^{(time)}$. This weight is given by the following function:

$$w_{i,c^*,t^*}^{(time)} = \left(\left(1 - \frac{|t[i] - t^*|}{1 + t_{c^*,max}} \right)^{\lambda_{c^*}} \right)^3, \tag{5}$$

where $t_{c^*,max}$ is the maximum time between t^* and all observation times for observations within the same region. The hyperparameter λ_c controls the smoothness of the smoothed residuals: a larger λ_c implies more smoothness (the weights diminish slowly over time), and a smaller λ_c less smoothness (the weights fall off quickly over time.) The value of λ_c for each country is chosen based on a measure of each country's data density (see Dicker et al. (2018) Supplemental Material page 20 for a full description).

The spatial and temporal weights were used to generate two sets of smoothed residuals for every country and year using two different methods as follows (see Dicker et al. (2018) page 16-17 in Supplementary material 1 for full details):

- The first set of smoothed residuals, which we denote $\hat{r}_{c,t}^{(\text{local linear})}$ is from a local linear fit to the weighted residuals (the authors describe it as similar to a Loess fit).
- The second set of smoothed residuals, which we denote $\hat{r}_{c,t}^{(\text{weighted average})}$ is calculated as an average of the weighted residuals $\hat{r}_i$.

The exact specification of the two fits is not clear to us as the authors do not provide mathematical descriptions. The two sets of smoothed residuals were combined together, with more weight going to the weighted average in data-sparse countries:

$$\hat{r}_{c,t}^{\text{smoothed}} = k_c \cdot \hat{r}_{c,t}^{(\text{local linear})} + (1 - k_c)\hat{r}_{c,t}^{(\text{weighted average})} \tag{6}$$

where

$$k_c = \frac{(\text{number of observed data points in country } c)}{(\text{number of observed data points in country } c) + (\text{number of years in country } c \text{ with no data})} \tag{7}$$

The smoothed residuals were added to $\hat{\eta}_i^{\text{predicted}}$ to yield a final set of predictions, which we denote $\hat{\eta}_{c,t}^{\text{smoothed}}$:

$$\hat{\eta}_{c,t}^{\text{smoothed}} = \hat{\eta}_{c,t}^{\text{predicted}} + \hat{r}_{c,t}^{\text{smoothed}} \tag{8}$$

Note that the smoothed residuals $\hat{r}_{c,t}^{\text{smoothed}}$ —with appropriate transformations —become the offsets $a_{c,t}$ in the model as written in the TMMP notation in Section 5.1 of the full paper.

Third Stage: GPR In the third modeling stage, a Gaussian process is used to produce final estimates. The observed data y_i are assumed to be normally distributed around the true value of the indicator on the log10-transformed scale:

$$\log_{10} y_i \mid \eta_{c[i],t[i]} \sim N(\log_{10} \eta_{c[i],t[i]}, s_i^2).$$

The observation variance s_i^2 is fixed depending on the data source. For example, for observations from complete vital registration systems, the only source of error is assumed to be from stochastic error, so s_i^2 is set to a stochastic error estimated using a binomial model. For other data sources various preprocessing steps were used to estimate the observation variance; see Dicker et al. (2018) Supplementary material page 19 for more information.

The latent true U5MR values from each country are smoothed via a Gaussian Process, again on the log10-transformed scale:

$$\log_{10} \boldsymbol{\eta}_c \mid \boldsymbol{\mu}_c, \boldsymbol{\Sigma}_c \sim MVN\left(\boldsymbol{\mu}_c, \boldsymbol{\Sigma}_c\right).$$

The mean of the multivariate normal distribution is set to the log10-transformed smoothed values from the second modeling stage: $\mu_{c,t} = \log_{10}\left(\hat{\eta}_{c,t}^{\text{smoothed}}\right)$. The covariance matrix $\boldsymbol{\Sigma}_c$ is derived from a Matérn autocovariance function with fixed hyperparameters. The hyperparameters of the Matérn covariance kernel are allowed to be different for each country to allow for varying levels of smoothness based on the amount and quality of data available in a country. See Dicker et al. (2018) page 20 in the Supplementary material 1 for a description of the data availability measure and a table giving the corresponding hyperparameter values.

UN-IGME model

The model used by the UN-IGME is referred to the 'B3' model, which refers to the fact that the model is a Bayesian, bias-adjusted B-Splines model (Alkema and New (2014)). The observed data, y_i, $i = 1, \ldots, N$ are derived from preprocessed raw data from vital registration systems, sample vital registration systems, surveys, and censuses. The observed data and are in terms of deaths per 1,000 live births ($y_i \in [0, 1000]$).

This model set-up also assumes that the observed data are normally distributed around the latent true population mean of the indicator:

$$\log y_i = \log(\eta_{c[i],t[i]}) + \delta_i,$$

where error δ_i is normally distributed with mean and variance depending on the data source, as described below. For complete vital registration systems, the error term is normally distributed with zero mean and a fixed stochastic variance v_i^2:

$$\delta_i \sim N(0, v_i^2).$$

For survey observations or data from other sources, the sampling variance is decomposed into a fixed sampling error and estimated non-sampling error. Systematic biases are also estimated, either as a linear trend for data sources with multiple observations or as an offset otherwise:

$$\delta_i \mid \Psi_i, \Sigma_i \sim N(\Psi_i, \Omega_i + v_i^2),$$

where Ψ_i is a systematic bias parameter and Ω_i is an estimated non-sampling error. For a complete description of how the systematic biases, sampling errors, and non-sampling errors were estimated, we refer interested readers to Section 3.2.1 of Alkema and New (2014).

The true U5MR values $\eta_{c,t}$ are smoothed via a cubic B-spline model:

$$\log(\eta_{c,t}) = \sum_{k=1}^{K_c} b_{c,k}(t)\alpha_{c,k},$$

where K_c are the number of knots in country c, $b_{c,k}$ is the kth spline value in country c, and $\alpha_{c,k}$ is the kth spline coefficient.

The spline coefficients during the observation period are parameterized as following a linear trend with random walk deviations away from that trend:

$$\alpha_{c,k} = \lambda_{c,0} + \lambda_{c,1}(k - K_c/2) + [\boldsymbol{D}'_{K_c}(\boldsymbol{D}_{K_c}\boldsymbol{D}'_{K_c})^{-1}\boldsymbol{\varepsilon}_c]_k$$

where $\lambda_{c,0}$ and $\lambda_{c,1}$ are the unknown level and slope parameters for the spline coefficients in country c and parameter vector $\boldsymbol{\varepsilon}_c = (\varepsilon_{c,1}, \ldots, \varepsilon_{c,Q_c})'$ contains the $Q_c = K_c - 2$ second-order differences in the spline coefficients, $\varepsilon_{c,q} = (\alpha_{c,q+2} - \alpha_{c,q+1}) - (\alpha_{c,q+1} - \alpha_{c,q})$ for $q = 1, \ldots, Q_c$; $[\boldsymbol{D}'_{K_c}(\boldsymbol{D}_{K_c}\boldsymbol{D}'_{K_c})^{-1}\boldsymbol{\varepsilon}_c]_k$ refers to the k-th element of vector $\boldsymbol{D}'_{K_c}(\boldsymbol{D}_{K_c}\boldsymbol{D}'_{K_c})^{-1}\boldsymbol{\varepsilon}_c$, with known difference matrix $\boldsymbol{D}_{K_c}$ (defined by $D_{K_c,i,i} = D_{K_c,i,i+2} = 1$, $D_{K_c,i,i+1} = -2$ and $D_{K_c,i,j} = 0$ otherwise). Vague priors were used for the $\lambda_{c,0}$'s and the $\lambda_{c,1}$'s. Second-order differences are penalized by imposing

$$\varepsilon_{c,q}|\sigma_c^2 \sim N(0, \sigma_c^2), \text{ for } q = 1, \ldots, Q_c,$$

where variance σ_c^2 determines the extent of smoothing; a smaller variance corresponds to smoother trajectories. A multilevel model is placed on the smoothing parameters, i.e. the standard deviation of $\varepsilon_{c,q}$, to share information between countries:

$$\log(\sigma_c)|\chi, \varphi_\sigma^2 \sim N(\chi, \varphi_\sigma^2),$$

where χ and φ_σ^2 refer to the mean and variance of the log-transformed standard deviations.

UN IGME uses B3 to produce estimates of U5MR up to the most recent year. A logarithmic pooling approach was used to produce estimates past the most recent observation year to combine country-specific posterior predictive distributions (PPDs) for changes in spline coefficients with a global PPD. This procedure was applied to modify the PPDs for $\alpha_{c,k}$ for $k = K_c, K_c + 1, \ldots, P_c$, where K_c and P_c refer to the indices of the most recent splines in the observation and projection periods respectively. The approach is summarized (leaving out indices referring to posterior samples) as follows:

$$\varepsilon_{c,K_c+a}|\Gamma_{c,K_c+a}, \Theta_{c,K_c+a} \sim N\left(\Gamma_{c,K_c+a}, \Theta_{c,K_c+a}\right), \text{ for } a \geq 0,$$

where

$$\Gamma_{c,k} = W \cdot G + (1 - W) \cdot \varepsilon_{c,k-1},$$
$$\Theta_{c,k} = W \cdot V + (1 - W) \cdot \Theta_{c,k-1},$$

with G and V equal to the median and variance of the estimates of past 2nd-order differences $\hat{\varepsilon}_{1:C,1:K_c}$'s respectively, and $\Theta_{c,K_c-1} = \sigma_c^2$. The overall pooling weight $0 \leq W \leq 1$ was chosen through an out-of-sample validation exercise.

Appendix S2

Properties of stationary smoothing models, with $r = 0$

When there is no differencing in the smoothing model ($r = 0$) then by construction the distribution of the smoothing terms is stationary: the unconditional first and second moments of $\boldsymbol{\epsilon}_c$ will not depend on time. As we will see shortly, this implies that in the absence of data the smoothing term will be centered around zero. This is important for understanding the behavior of the model in projections: the smoothing model will contribute to uncertainty in the projections, but since it will eventually revert to mean zero the projections

will be centered around the other process model components. As we will see in the next section, this is not true when $r > 0$ because the smoothing terms are no longer stationary.

To make our understanding of the behavior of these stationary smoothing models more precise and to facilitate comparison across smoothing models, we can look at their conditional distributions. This helps particularly in exploring the implied projections of each smoothing model given a last observed data point.

Suppose we have observed data up to time t^*, and let $\boldsymbol{\epsilon_{t^*}} = [\epsilon_1, \cdots, \epsilon_{t^*}]$. We set $\boldsymbol{B} = \boldsymbol{I}$ to simplify the analysis, which means $\boldsymbol{\epsilon} = \boldsymbol{\delta}$ (the two are interchangeable, and we drop the subscript c for simplicity). Similarly define $\boldsymbol{\delta}_{t^*} = [\delta_1, \cdots, \delta_{t^*}]$. We placed the restriction that $\boldsymbol{\delta}$ be multivariate normally distributed, which means there is a closed-form solution for the distribution of $\boldsymbol{\delta}$ conditional on the observed data. The conditional mean of δ_t given $\boldsymbol{\delta}_{t^*}$ for $t > t^*$ is given by

$$\mathrm{E}[\delta_t \mid \boldsymbol{\delta}_t^*] = L^\top \Sigma^{-1} \boldsymbol{\delta}_{t^*},$$

where L is the Gram matrix derived from the covariances between δ_t and every element of $\boldsymbol{\delta}_{t^*}$ ($L_i = s(t, i)$ for $i = 1, \cdots, t^*$), and Σ is the Gram matrix derived from the covariances between every pair in $\boldsymbol{\delta}_{t^*}$. We restricted the covariance function of the smoothing model to depend only differences in time, and that it goes to zero as the differences in time grows. This ensures that the conditional mean is guaranteed to converge to zero as $t \to \infty$. The manner in which the conditional means converge is determined by the specific covariance function. For example, the sparsity of Σ^{-1} for an AR(1) kernel makes it straightforward to derive the conditional distribution:

$$\mathrm{E}[\delta_t \mid \boldsymbol{\delta}_{t^*}] = \delta_{t*} \cdot \rho^{(t-t^*)}.$$

In projections, an AR(1) process depends only on the last observed value (δ_{t^*}), converging back to zero as $\rho^{(t-t^*)}$ converges to zero. This is a consequence of the sparsity of the precision matrix for the AR(1) covariance. Covariance functions that yield non-sparse precision matrices do not lead to conditional means with simple forms like the AR(1) process. This leads to more complex behavior: for example, the squared exponential kernel can project trends in the previously observed data before returning to zero. Figure 1 compares the conditional behavior of several covariance functions.

Non-stationary smoothing models, with $r > 0$

When the degree of differencing is greater than zero ($r > 0$) then the resulting smoothing models are not stationary (their unconditional first and second moments will depend on time). However, differencing can yield a stationary process. For example, a RW(1) process, with

$$\delta_t | \delta_{t-1}, \sigma \sim N(\delta_{t-1}, \sigma^2),$$

after one level of differencing becomes

$$\triangle \delta_t \mid \sigma \sim N(0, \sigma^2).$$

Therefore within our framework the RW(1) model can be expressed as $r = 1$ (one level of differencing to yield a stationary process) and $\boldsymbol{\Sigma}_c = \sigma^2 \boldsymbol{I}$ (the covariance matrix of the differenced stationary process is diagonal). Similarly, a RW(2) process after two levels of differencing is stationary, so we describe it as $r = 2$ and $\boldsymbol{\Sigma}_c = \sigma^2 \boldsymbol{I}$. Autoregressive integrated moving average (ARIMA(p,d,q)) models are obtained by setting $r = d$ and using the respective autocovariance function in $\boldsymbol{\Sigma}_c$. For example, the autoregressive integrated ARI(1,1) model is given by $r = 1$ and the AR(1) covariance function.

Unconstrained non-stationary smoothing processes can be written according to the TMMP process model specification by a reparametrization of such processes into a zero-constrained process and a structural component. For example, an unconstrained RW(1) ($d = 1$) for years $\mathcal{K}_{1,c}$ can be reparametrized into one that includes a sum-to-zero constraint by introducing as parameters the mean of the process and the deviations away from the mean. This parametrization is most clearly expressed using a first order differencing matrix $\boldsymbol{D}$ with $D_{i,i} = -1$, $D_{i,i+1} = 1$, and is zero everywhere else. With $\boldsymbol{D}$ and $\mathcal{K}_{1,c} = \{1, 2, \ldots, t^*\}$, we can write

$$\boldsymbol{\delta}_{t^*} = \alpha_0 + [\boldsymbol{D}'(\boldsymbol{D}\boldsymbol{D}')^{-1}] \boldsymbol{\gamma}_{t^*-1},$$

where $\alpha_0 = 1/t^* \sum_t \delta_t$ and $\gamma_t = \delta_t - \delta_{t-1}$ for $t = 2, ..., t^*$. Hence, an unconstrained RW(1) process δ_t can be rewritten as the combination of a smoothing process γ with $\sum_{t \in \mathcal{K}_{1,c}} \triangle_d \gamma_{c,t} = 0$ and a systematic component $g_3(t, \alpha_c) = \alpha_0$ for $t \in \mathcal{K}_{d,c}$. Moving the intercept from the smoothing component to the systematic component also helps to clarify the behavior of the model: an overall level during the period associated with $\mathcal{K}_{1,c}$ is estimated as a systematic trend, and deviations are left to the smoothing model. Similarly, an unconstrained RW(2) process can be reparametrized into the mean and rate of change of the process during sets of years $\mathcal{K}_{1,c}$ and $\mathcal{K}_{2,c}$.

Non-stationary smoothing models do not necessarily revert to mean zero in the absence of data, unlike the models we saw in the previous section. As such, non-stationary models can influence the trend of projections. The RW(1) model will extend forward the last observed data point, and the RW(2) model will extend forward a linear trend based on $\triangle\delta_t \mid \sigma, \triangle\delta_{t-1} \sim N(\triangle\delta_{t-1}, \sigma^2)$. The behavior of projections will therefore be determined by the interplay between the smoothing and the covariate and systematic process model components that extend past $\mathcal{K}_{d,c}$.

Appendix S3

The following tables are templates for specifying models that fall within the TMMP framework. Four example specifications are provided for each of the additional examples described in the main text.

	**FPEM**
Citation	Cahill et al. (2018)
$\eta_{c,t}$	total contraceptive use rate
$g_1(\cdot)$	logit
Process model formula	$\text{logit}(\eta_{c,t}) = g_3(\cdot) + \epsilon_{c,t}$
**Covariate Component**	
$g_2(\cdot)$	.
Covariates	.
**Systematic Component**	
$g_3(\cdot)$	Logistic curve:<br>$g_3(\cdot) = \Omega_c$ when $t = t^*$, and for $t > t^*$:<br>$g_3(\cdot) = \text{logit}\left(\eta_{c,t-1}\right) + \delta_{c,t} =$<br>$\begin{cases} \text{logit}\left(\tilde{P}_c \cdot \text{logit}^{-1}\left(\text{logit}\left(\frac{\eta_{c,t-1}}{\tilde{P}_c}\right) + \omega_c\right)\right), & \text{when } \eta_{c,t-1} < \tilde{P}_c \\ \text{logit}\left(\eta_{c,t-1}\right), & \text{otherwise,} \end{cases}$<br>where $\boldsymbol{\alpha}_c = \left\{\tilde{P}_c, \omega_c, \Omega_c\right\}$.
$\boldsymbol{\alpha}_c$	$\tilde{P}, \omega_c, \Omega_c$
**Offsets**	
$a_{c,t}$	.
**Stochastic smoothing Component**	
$\boldsymbol{B}$	$\boldsymbol{B} = \boldsymbol{I}$
$s(t_1, t_2)$	AR(1); $s(t_1, t_2) = \frac{\rho^{\lvert t_1 - t_2 \rvert}}{\sigma^2(1-\rho^2)}$
r	0
$\mathcal{K}_{d,c}$	.

**Parameter Estimation**	
Fixed	·
Vague Priors	·
Informative Priors	·
Hierarchical model	systematic parameters $\tilde{P}, \omega_c, \Omega_c$
Hierarchical distribution π	normal
Number of levels in hierarchy	$\tilde{P}$: 1<br>ω_c: 3<br>Ω_c, developing countries: 3<br>Ω_c, developed countries: 1
Hierarchical groupings	$\tilde{P}$: countries within world<br>ω_c: countries within sub-region, region world<br>Ω_c, developing countries: countries within sub-region, region, world<br>Ω_c, developed countries: countries within world
**Projections**	
Projections	·

Table 1: TMMP specification for the Family Planning Estimation Model (Cahill et al., 2018).

	**NMR**
Citation	Alexander and Alkema (2018)
$\eta_{c,t}$	NMR / (U5MR - NMR)
$g_1(\cdot)$	log
Process model formula	$\log(\eta_{c,t}) = g_2(\cdot) + \epsilon_{c,t}$
**Covariate Component**	
$g_2(\cdot)$	$\beta_{c,0} + \beta_1 \log(X_{c,t} - \log(\beta_2))\mathbf{1}_{[X_{c,t} > \beta_2]}$
Covariates	U5MR
**Systematic Component**	
$g_3(\cdot)$	·
$\boldsymbol{\alpha}_c$	·
**Offsets**	
$a_{c,t}$	·
**Stochastic smoothing Component**	
$\boldsymbol{B}$	$B_{c,t,k} = b_{c,k}(t) =$ cubic B-splines
$s(t_1, t_2)$	independent $k(t_1, t_2) = \sigma^2 1(t_1 = t_2)$
r	1

$\mathcal{K}_{d,c}$	$\{1, \ldots, K_c\}$
**Parameter Estimation**	
Fixed	·
Vague Priors	regression coefficients
Informative Priors	·
Hierarchical model	smoothing parameters
Hierarchical distribution π	normal
Number of levels in hierarchy	1
Hierarchical groupings	countries within world
**Projections**	
Projections	·

Table 2: TMMP specification for the Neonatal Mortality Rate model (Alexander and Alkema, 2018).

	**Bmat**
Citation	Alkema et al. (2017)
$\eta_{c,t}$	proportion of non-AIDS deaths that are maternal among women of reproductive age
$g_1(\cdot)$	log
Process model formula	$\log(\eta_{c,t}) = g_2(\cdot) + \epsilon_{c,t}$
**Covariate Component**	
$g_2(\cdot)$	$\beta_{c,0} + \sum_k X_{c,t,k}\beta_k$
Covariates	log(GDP), log(GFR), SAB
**Systematic Component**	
$g_3(\cdot)$	·
$\boldsymbol{\alpha}_c$	·
**Offsets**	
$a_{c,t}$	·
**Stochastic smoothing Component**	
$\boldsymbol{B}$<br>$\boldsymbol{B} = \boldsymbol{I}$	$B_{c,k} = b_{c,k}(t) =$ cubic B-splines
$s(t_1, t_2)$	ARMA(1,1)
r	1
$\mathcal{K}_{d,c}$	$\{1990\}$

Parameter Estimation	
Fixed	·
Vague Priors	regression coefficients
Informative Priors	·
Hierarchical model	regression intercepts<br>smoothing parameters
Hierarchical distribution π	intercept: normal<br>smoothing: truncated normal
Number of levels in hierarchy	intercept: 2<br>smoothing: 1
Hierarchical groupings	intercept: countries within region within world<br>smoothing: countries within world
**Projections**	
Projections	·

Table 3: TMMP specification for Bmat (Alkema et al., 2017).

	**Mortality**
Citation	Alexander et al. (2017)
$\eta_{c,t}$	age-specific mortality
$g_1(\cdot)$	log
Process model formula	$\log(\eta_{c,t}) = g_2(\cdot) + \epsilon_{c,t}$
**Covariate Component**	
$g_2(\cdot)$	$\sum_k X_{a,k}\beta_{c,t,k}$
Covariates	$X_{k,a}$ is the kth principal component of the mortality schedule for age group a
**Systematic Component**	
$g_3(\cdot)$	·
$\boldsymbol{\alpha}_c$	·
**Offsets**	
$a_{c,t}$	·
**Stochastic smoothing Component**	
$\boldsymbol{B}$	$\boldsymbol{B} = \boldsymbol{I}$
$s(t_1, t_2)$	independent
r	0
$\mathcal{K}_{d,c}$	·
**Parameter Estimation**	

Fixed	·
Vague Priors	·
Informative Priors	·
Hierarchical model	regression coefficients, smoothing parameters
Hierarchical distribution π	normal
Number of levels in hierarchy	1
Hierarchical groupings	counties within state
**Projections**	
Projections	·

Table 4: TMMP specification for the age-specific mortality model (Alexander et al., 2017).

References

M. Alexander and L. Alkema. Global estimation of neonatal mortality using a Bayesian hierarchical splines regression model. *Demographic Research*, 38:335–372, Jan. 2018. ISSN 1435-9871. doi: 10.4054/DemRes.2018.38.15. URL `https://www.demographic-research.org/volumes/vol38/15/`.

M. Alexander, E. Zagheni, and M. Barbieri. A Flexible Bayesian Model for Estimating Subnational Mortality. *Demography*, 54(6):2025–2041, Dec. 2017. ISSN 0070-3370. doi: 10.1007/s13524-017-0618-7. URL `https://www.ncbi.nlm.nih.gov/pmc/articles/PMC5948000/`.

L. Alkema and J. R. New. Global estimation of child mortality using a Bayesian B-spline Bias-reduction model. *The Annals of Applied Statistics*, 8(4):2122–2149, Dec. 2014. ISSN 1932-6157. doi: 10.1214/14-AOAS768. URL `http://projecteuclid.org/euclid.aoas/1419001737`.

L. Alkema, S. Zhang, D. Chou, A. Gemmill, A.-B. Moller, D. M. Fat, L. Say, C. Mathers, and D. Hogan. A Bayesian approach to the global estimation of maternal mortality. *The Annals of Applied Statistics*, 11(3):1245–1274, Sept. 2017. ISSN 1932-6157. doi: 10.1214/16-AOAS1014. URL `https://projecteuclid.org/euclid.aoas/1507168829`.

N. Cahill, E. Sonneveldt, J. Stover, M. Weinberger, J. Williamson, C. Wei, W. Brown, and L. Alkema. Modern contraceptive use, unmet need, and demand satisfied among women of reproductive age who are married or in a union in the focus countries of the Family Planning 2020 initiative: a systematic analysis using the Family Planning Estimation Tool. *The Lancet*, 391(10123):870–882, Mar. 2018. ISSN 0140-6736. doi: 10.1016/S0140-6736(17)33104-5. URL `http://www.sciencedirect.com/science/article/pii/S0140673617331045`.

D. Dicker, G. Nguyen, D. Abate, and other GBD 2017 Mortality Collaborators. Global, regional, and national age-sex-specific mortality and life expectancy, 1950–2017: a systematic analysis for the Global Burden of Disease Study 2017. *The Lancet*, 392(10159):1684–1735, Nov. 2018. ISSN 01406736. doi: 10.1016/S0140-6736(18)31891-9. URL `https://linkinghub.elsevier.com/retrieve/pii/S0140673618318919`.

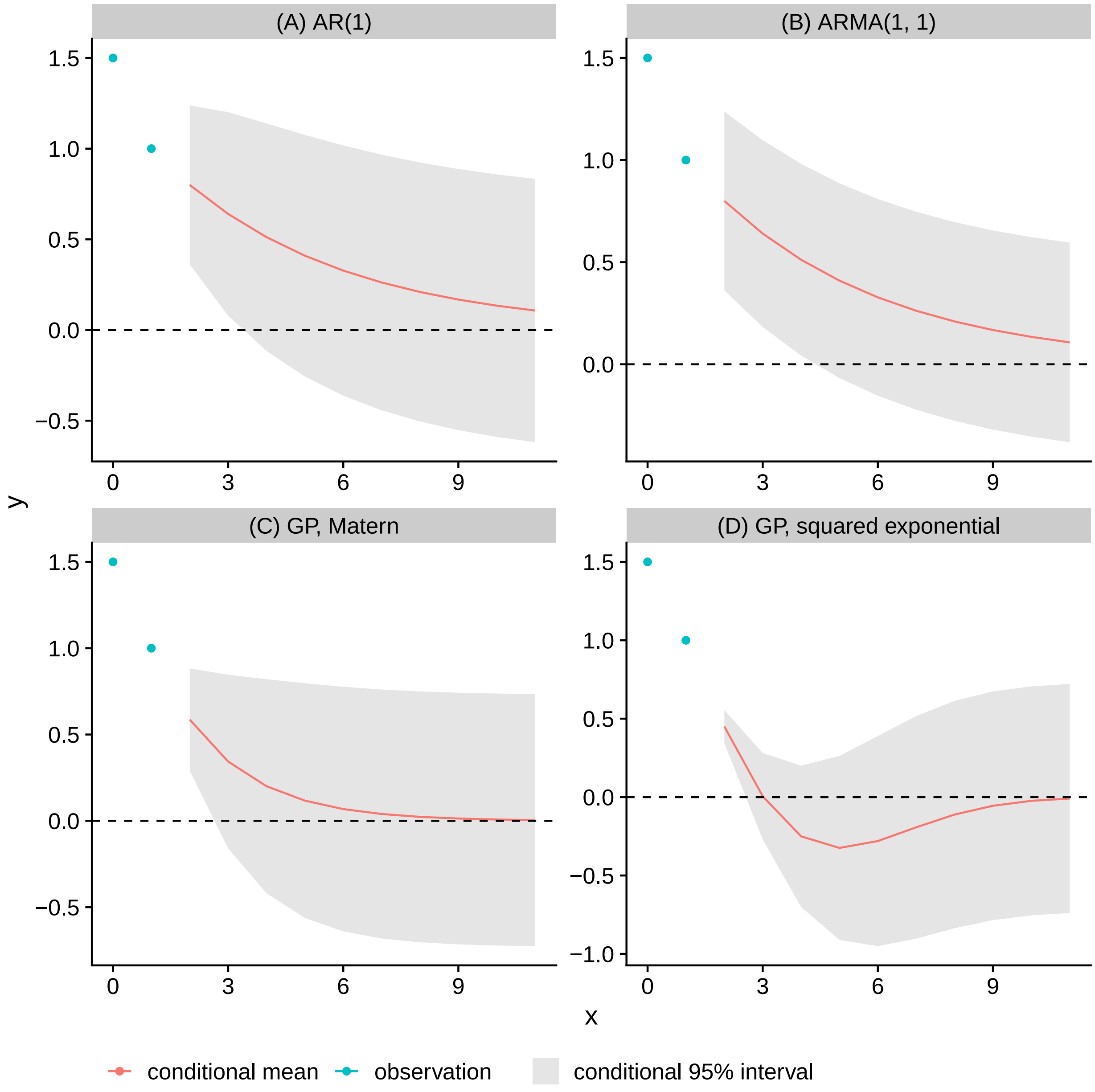

Figure 1: Comparison of the conditional distributions of four smoothing models based on two observed data points. The parameters for the smoothers are: (A) AR(1), $\rho = 0.8, \kappa^2 = 0.05$. (B) ARMA(1, 1), $\rho = 0.8$, moving average parameter $\theta = 0.8$, $\kappa^2 = 0.05$. (C) GP, Matérn, $\kappa^2 = 0.05/(1-\rho^2), \rho = 0.8, \nu = 3/2, \ell = 3$. (D) GP, squared exponential, $\kappa^2 = 0.05/(1-\rho^2), \rho = 0.8, \ell = 3$.
